# Unravelling the metabolomic diversity of pigmented and non-pigmented traditional rice from Tamil Nadu, India

**DOI:** 10.1186/s12870-024-05123-3

**Published:** 2024-05-15

**Authors:** Venkatesan Subramanian, Udhaya Nandhini Dhandayuthapani, Senthilraja Kandasamy, Jidhu Vaishnavi Sivaprakasam, Prabha Balasubramaniam, Mohan Kumar Shanmugam, Sriram Nagappan, Subramanian Elangovan, Umesh Kanna Subramani, Kumaresan Palaniyappan, Geethalakshmi Vellingiri, Raveendran Muthurajan

**Affiliations:** 1 Directorate of Research, Tamil Nadu Agricultural University, Coimbatore, Tamil Nadu 641 003 India; 2 Centre of Excellence in sustaining Soil Health, Anbil Dharmalingam Agricultural College & Research Institute, Trichy, Tamil Nadu 620 027 India; 3 Amrita School of Agricultural Sciences, Tamil Nadu Coimbatore, 642 109 India; 4 Department of Renewable Energy Engineering, Tamil Nadu Agricultural University, Coimbatore, Tamil Nadu 641 003 India; 5 Agro-Climatic Research Centre, Tamil Nadu Agricultural University, Coimbatore, Tamil Nadu 641 003 India; 6 Krishi Vigyan Kendra, Madurai, Tamil Nadu 625 104 India; 7 Office of the Vice Chancellor, Tamil Nadu Agricultural University, Coimbatore, Tamil Nadu 641 003 India; 8 Agribusiness Development, Tamil Nadu Agricultural University, Coimbatore, Tamil Nadu 641 003 India

**Keywords:** Traditional rice variety, Metabolite biomarkers, Principal compound analysis, Gas chromatography-mass spectrometry, SDG 2, KEGG pathway, Univariate and multivariate analysis

## Abstract

Rice metabolomics is widely used for biomarker research in the fields of pharmacology. As a consequence, characterization of the variations of the pigmented and non-pigmented traditional rice varieties of Tamil Nadu is crucial. These varieties possess fatty acids, sugars, terpenoids, plant sterols, phenols, carotenoids and other compounds that plays a major role in achieving sustainable development goal 2 (SDG 2). Gas-chromatography coupled with mass spectrometry was used to profile complete untargeted metabolomics of Kullkar (red colour) and Milagu Samba (white colour) for the first time and a total of 168 metabolites were identified. The metabolite profiles were subjected to data mining processes, including principal component analysis (PCA), Orthogonal Partial Least Square Discrimination Analysis (OPLS-DA) and Heat map analysis. OPLS-DA identified 144 differential metabolites between the 2 rice groups, variable importance in projection (VIP) ≥ 1 and fold change (FC) ≥ 2 or FC ≤ 0.5. Volcano plot (64 down regulated, 80 up regulated) was used to illustrate the differential metabolites. OPLS-DA predictive model showed good fit (R2X = 0.687) and predictability (Q2 = 0.977). The pathway enrichment analysis revealed the presence of three distinct pathways that were enriched. These findings serve as a foundation for further investigation into the function and nutritional significance of both pigmented and non-pigmented rice grains thereby can achieve the SDG 2.

**Supplementary Information:**

The online version contains supplementary material available at 10.1186/s12870-024-05123-3.

## Background

Rice (*Oryza sativa* L) is a primary dietary component for more than half of the global population and ranks as the second largest cultivated cereal crop across the globe [1, 2]. SDG 2 specifically addresses food, aiming to “end hunger, achieve food security and im-proved nutrition and promote sustainable agriculture.” However, a number of other goals also address issues facing the food system.

The realization has emerged in recent years that traditional rice varieties constitute a valuable gene pool for features that may support modern rice varieties ability to adapt to climate change, particularly in light of the phenomenon of climate change. After COVID 19 Pandemic, the eating behaviour of the Indian people especially the Tamilians were changed drastically. This mainly highlighted the importance of rebuilding the immune system through nutrition rich diet balances [3].

Traditional rice can be categorized into two types: pigmented and non-pigmented. Non-pigmented rice is consumed by approximately 85% of the world’s population, whereas pigmented rice has traditionally been enjoyed primarily in China, Japan, and Korea due to its distinct flavor and perceived health benefits [4].

The rice grain contains metabolites that exhibit protective properties against human diseases when consumed through the diet. These metabolites also contribute positively to the immune system. In recent years, there has been a growing interest in pigmented rice varieties, with red rice in particular garnering attention due to its bioactive compounds. These compounds have been found to possess superior antioxidant, anti-inflammatory, antitumor, and hypoglycemic effects as supported by various studies [5–9]. In addition to its various health advantages, including other benefits as mentioned by [10, 11], it is noteworthy that rice with darker shades holds properties beyond those found in light-colored varieties.

The presence of distinct polyphenol subgroups in whole grain rice of different colors, which have the potential to positively influence human well-being [11, 12]. The primary phenolics found in red rice varieties include ferulic acid, *p*-coumaric acid, and vanillic acid [12] which is also reported in this study. Studies have suggested that *p*-coumaric acid and vanillic acid may play a crucial role in the antioxidant activity of red rice [13]. Furthermore, existing research indicates a notable positive correlation between phenolic components and antioxidation [12, 14–16].

The predominant focus of research on pigmented rice has been on the relationship between anthocyanins and antioxidants, as well as its nutritional properties. This is because rice is widely acknowledged as a functional food in various Asian countries, known for its numerous reported health advantages [17]. Conversely, Red rice (another type of pigmented rice) boasts elevated levels of proanthocyanidins and other phenolics [18, 19]. Additionally, it has been observed that pigmented rice exhibits a higher level of antioxidant activity compared to non-pigmented rice. The degree of pigmentation also plays a role, with darker pigmentation indicating a greater presence of flavonoids and thus stronger antioxidant properties [12, 20, 21].

These cultivars have rich and varied nutrient profiles including antioxidants, which greatly contribute to global food security and nutrition. Thereby, traditional rice is very compatible with SDG 2’s objective of guaranteeing that everyone has access to safe and nourishing food because of their ability to prevent malnutrition and address certain nutrient deficits. Their production also promotes resilience to climate change, sustainable farming methods, and the preservation of agricultural biodiversity.

Extensive research has explored the different metabolites found in both types of rice around the world. However, until now, there have been few published studies on the metabolomics of pigmented and non-pigmented traditional rice varieties of Tamil Nadu. Hence, it is important to highlight the absence of comprehensive metabolic profiling that encompasses primary and secondary metabolites across various cultivars of pigmented and non-pigmented traditional rice. Additionally, there is a need to comprehend the metabolic networks that connect extensive datasets from metabolite profiling with metabolic pathways.

The present study employed a gas chromatography-mass spectrometry (GC-MS/MS) metabolomics technique, specifically using triple quadrupole mass spectrometry. This technique was utilized to explore and quantify primary and secondary metabolites in pigmented and non-pigmented traditional rice varieties, namely Kullakar and Milagu Samba. The metabolite profiling of Kullakar and Milagu Samba is the first study of its kind. The analysis involved chemometric methods like principle component analysis (PCA) and partial least square discrimination analysis (PLS-DA) to effectively classify the samples based on their diversity. To categorize the functional metabolites that offer health benefits from traditional rice varieties, we conducted hierarchical cluster analysis and metabolic pathway identification. The results of this study will provide a valuable theoretical foundation for developing functional foods using traditional rice.

## Materials and methods

**Fig. 1:**
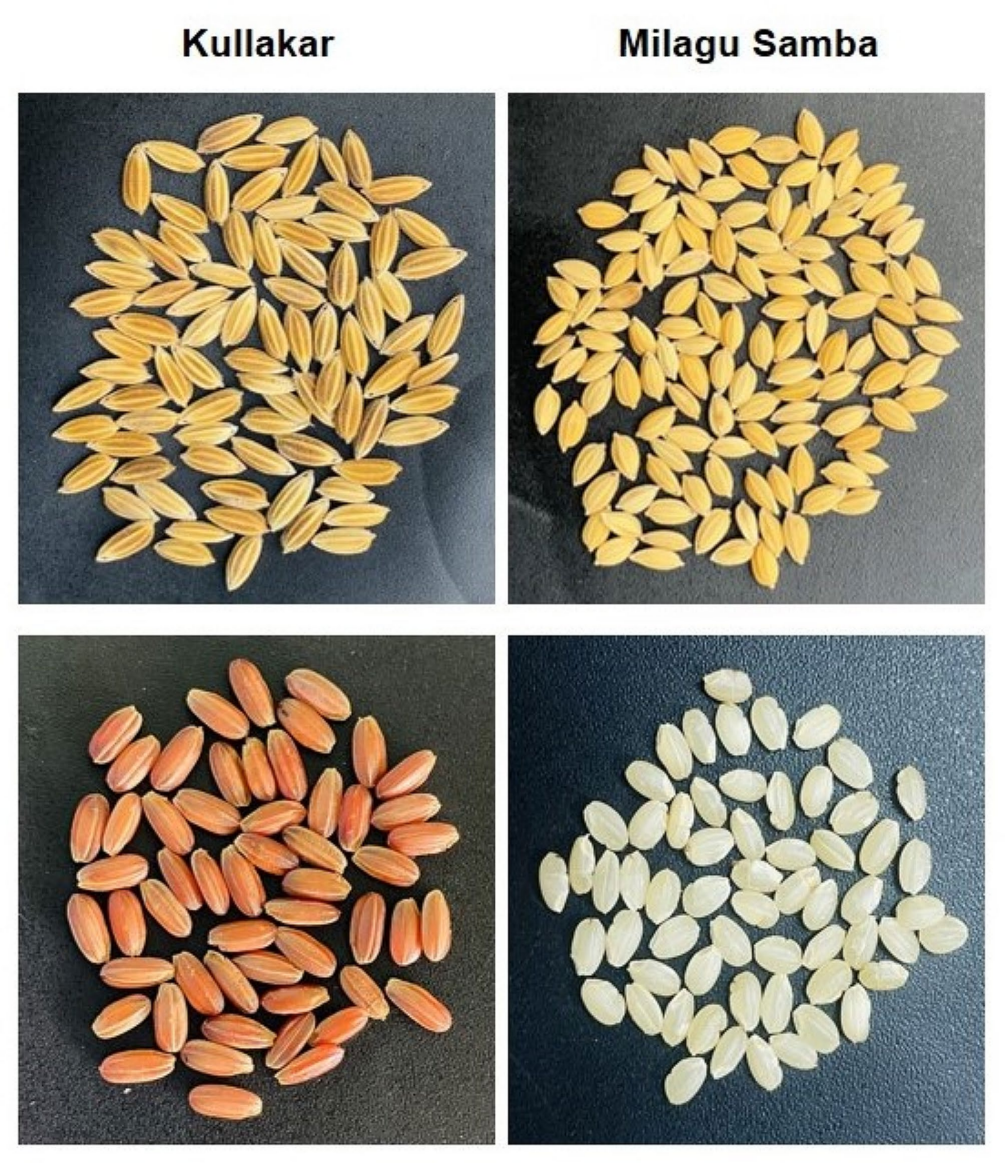
Photographs illustrating the pigmented and non-pigmented traditional rice

Two rice cultivars were chosen based on their pericarp colour: Red rice (Kullakar) and white rice (Milagu Samba) (Fig. 1; Table 1). Prior to collecting the plant material, we have obtained legal permission from the Director of Research at Tamil Nadu Agricultural University in Coimbatore, Tamil Nadu, India. The collection process adheres to all institutional, national and international guidelines and legislation. The samples included in this study were exclusively collected from the farmers. The mentioned traditional varieties were cultivated by the farmers of Thanjavur district during the *Samba* season (July-December, 2021) by adopting the required agronomic practices. Following cultivation, the grains were sun-dried to achieve a water content of approximately 10–11%, and subsequently stored in darkness at a temperature of 4 °C until further use. Prior to analysis, each sample underwent manual dehulling to obtain brown rice, while any broken grains were discarded. To ensure accuracy and reliability of results, three biological replicates were conducted for each rice cultivar.

**Table 1:** Detailed agronomic characteristics of the selected traditional rice

Name of the Variety	Kullakar	Milagu Samba
Origin	Tamil Nadu, India	Tamil Nadu, India
Pedigree	Unknown	Unknown
Duration (Days)	120–125	135–140
Average height (cm)	110	120
Number of grains per ear head	100–110	150–170
Yield of grain (kg acre^− 1^)	1400	1500
1000 grain weight (g)	25	20
Colour of pericarp	Red	White

### Sample preparation and extraction

To begin the analysis, a 20 ml centrifuge tube was utilized. Inside the tube, one gram of finely ground rice sample was carefully placed. To create a suitable solution, 10 ml of HPLC grade ethanol was added to the tube. The mixture in the tube underwent vortexing using a LABOID (International instrument from Himachal Pradesh, India) at a speed of 2000 rpm for a duration of ten minutes. After vortexing, the mixture was subjected to centrifugation for 20 min at a speed of 5000 rpm [22]. Following centrifugation, the supernatant obtained from the process underwent concentration using a rotary evaporator and subsequently filtered utilizing a PVDF syringe filter with a pore size of 0. 2 μm. The resulting filtrate was then stored in an air-tight glass vial at temperatures of 4 °C in preparation for further chromatographic analysis.

### Chromatography Condition and Analysis

The ethanolic extract that had undergone filtration was analyzed for metabolites using a Thermo Fisher ISQ triple quadrupole gas chromatograph - mass spectrometer, specifically the Thermo Fisher TSQ 8000 Duo Triple Quadrupole GC-MS/MS. The gas chromatograph (GC) was equipped with a fused silica capillary column DB-5 measuring 30 m in length and 0. 25 mm in internal diameter, with a film thickness of 0. 25 μm. Helium gas was utilized as the carrier gas at a flow rate of 1.0 ml/min. Subsequently, a volume of 1 ml from the specimen was preserved in a screw-top vial of 2 ml capacity, which was then loaded into an auto-injector. A minute quantity of 1 µl from the sample was injected in split mode (ratio: 1:10). The temperature of both the detector and injector were maintained at 250^◦^C throughout the process. The oven temperature was programmed to increase gradually, starting at 70◦C for a duration of 15 min, followed by a rapid rise to 280^◦^C at a rate of 30^◦^C per minute. Afterward, it remained constant for another ten minutes be-fore being lowered to 250^◦^C at a rate of ten degrees per minute. The mass spectrometer set-tings consisted of operating in full scan mode with electron impact spectra at an energy level of 70 eV. Additionally, the ion source temperature was set at 260^◦^C while the trans-mission line temperature remained steady at 280^◦^C. The range of the mass scan, measured in mass-to-charge ratio (*m/z*), was set between 50 and 650 atomic mass units (amu). A solvent delay of 3 min was implemented [23]. The identification of bioactive molecules involved comparing their mass spectra with the NIST 08 Mass Spectra Library, which is maintained by the National Institute of Standards and Technology. The name, molecular weight, and structure of the identified molecules were determined using data-bases such as NIST, Pub Chem and HMDB.

### Statistical analysis

The experiments were conducted in triplicate and the metabolites were annotated using the Kyoto Encyclopedia of Genes and Genomes (KEGG) database and the human metabolome database (HMDB). To analyze the metabolites, both univariate and multivariate analysis techniques were employed using the R package-based Metabo Analyst 5. 0 [24]. Specifically, an OPLS-DA model was utilized to compare the metabolic characteristics of pigmented and non-pigmented rice varieties. Prior to analysis, the metabolite data underwent normalization and auto scaling procedures. The criteria for screening differential metabolites involved setting the variable importance in the projection (VIP) value to be greater than or equal to 1 in the OPLS-DA model, and also requiring an absolute Log2FC (fold change) value of at least 1. VIP metabolites were subjected to Debiased Sparse Partial Correlation algorithm (DSPC) and pathway map network in Cytoscape software. Venn diagrams were utilized to visually represent the count of these differential metabolites. Furthermore, pathways containing metabolites that exhibited significant regulation were subjected to a metabolite sets enrichment analysis (MSEA). The significance of these pathways was evaluated using p-values derived from hypergeometric tests.

## Results

### Metabolite detection

The metabolite profiles of samples were systematically analyzed pigmented (Kullakar) and non-pigmented (Milagu Samba) rice for the first time in this research work. A comprehensive non targeted metabolite analysis of pigmented and non-pigmented rice was conducted using GC-MS/MS revealed total of 168 metabolites (Table S1, Fig. S1 & S2). Precisely, Kullakar exhibited 114 metabolites whereas non-pigmented Milagu Samba dis-closed with 103 metabolites with 49 metabolites being shared commonly between 2 varieties. Identified 168 metabolites includes 11 Benzene and substituted derivatives, 13 each under Prenol lipids and Saturated hydrocarbons, 19 separately by Organooxygen compounds and Steroids and steroid derivatives and 62 Fatty Acyls. Biological classes namely Acyl halides, Carboxylic acids and derivatives, Dihydrofurans, Phenols, Pyridines and derivatives and Unsaturated hydrocarbons consists of 2 metabolites under each category. The remaining 19 classes shares one metabolite individually.

**Fig. 2:**
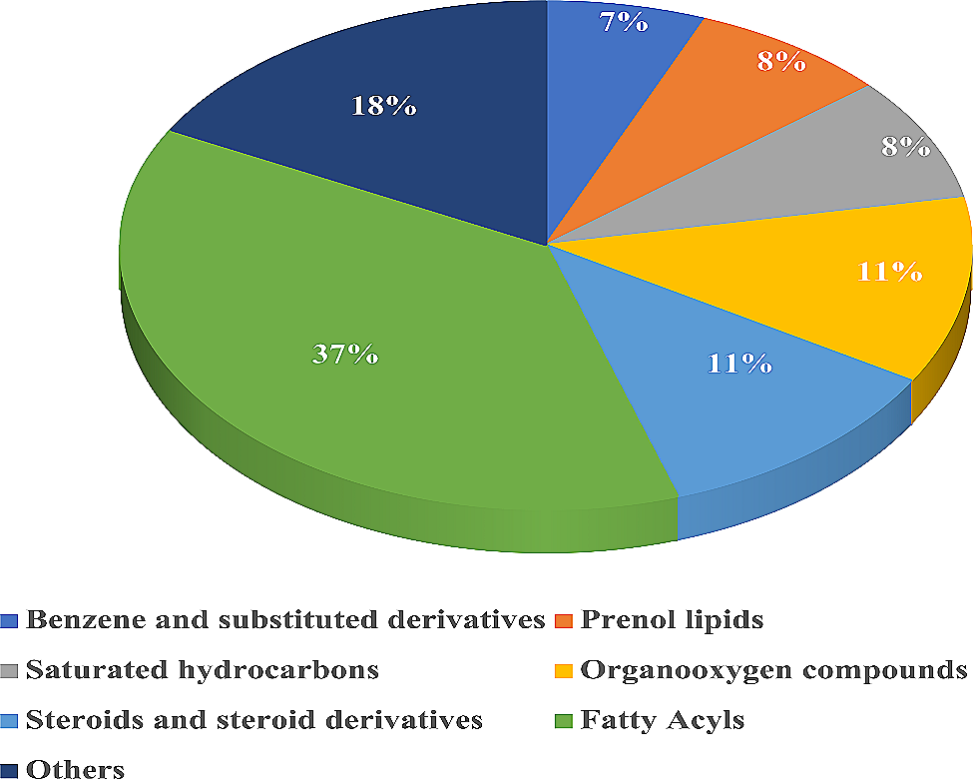
The provided pie chart illustrates the distribution of identified metabolites across various biological classes, as per the classifications of the Human Metabolome Database (HMDB)

Identified metabolites were categorized into 32 significant biological classes. These covered of Benzene and substituted derivatives (6.63%), Prenol lipids (7.83%), Saturated hydrocarbons (7.83%), Organooxygen compounds (11.40%), Steroids and steroid derivatives (11.40%) and Fatty Acyls (37.3%) (Fig. 2). Classes such as Acyl halides, Carboxylic acids and derivatives, Dihydrofurans, Phenols, Pyridines and derivatives and Unsaturated hydrocarbons shares 1.20% each.

**Fig. 3:**
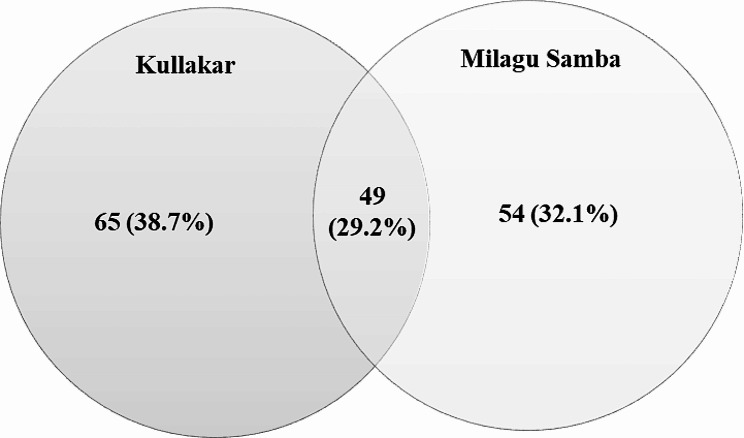
Venn diagram can be employed to visually represent the shared and distinct metabolites between pigmented and non-pigmented varieties of rice

A Venn diagram was constructed to exemplify the overlapping and differed metabolites between Kullakar and Milagu Samba (Fig. 3; Table S2). In the pairwise comparison, 49 metabolites were found to be overlapped that includes majorly fatty acids, steroids, sugars, terpenes and alkanes. Venn diagram exhibited 65 metabolites were particular to Kullakar and 54 to Milagu samba.

### Principal component analysis

**Fig. 4:**
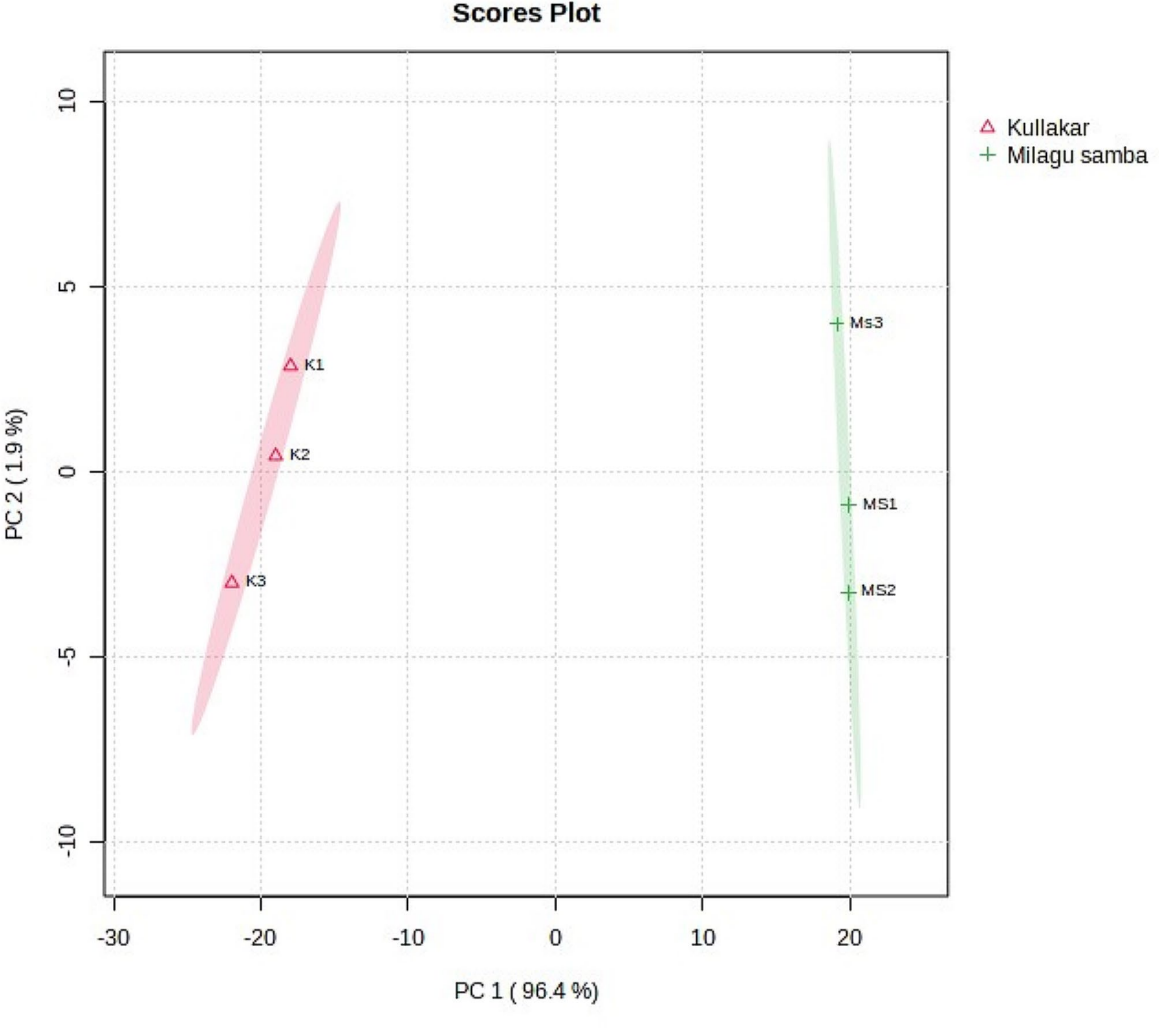
Score plot of PCA

A complete multivariate statistical analysis called PCA was performed on two different coloured traditional rice to explicate the dissimilarities in their metabolite composition. The principal components (PCs) that exhibited eigen value of greater than one was held in the study. Selected PCs in the score diagram (Fig. 4) clearly indicates the metabolite loadings of Kullakar on the negative and the non-pigmented on positive side which undoubtedly demonstrates the pigmentation based discernible variation between the samples. The unsupervised classification extracted two PCs explaining 98.30% of total variation (Table S3; Fig. S3).

The first PC alone described 96.4% variation that is correlated to 21 metabolites such as (3beta,4alpha,5alpha,9beta)-4,14-Dimethyl-9,19-cycloergost-24-en-3-ol, (9*Z*,11 S,16 S)-1-Acetoxy-9,17-octadecadiene-12,14-diyne-11,16-diol, 6-Octadecenoic acid, Acetylhydrazine, alpha-Sitosterol, Benzothiazole, Bovinic acid, Cholest-5-ene, Choles-tan-3-ol, cis-Vaccenic acid, Clionasterol, Dihydrobrassicasterol, Isofucosterol 3-O-[6-O-(9,12-Octadecadienoyl)-b-D-glucopyranoside], Lignocerane, Linoleic acid, Me-thyl hexadecanoic acid, Palmitic acid, Paullinic acid, Pentadecanoic acid, Stigmasterol and trans-12-Octadecenoic acid. The second PC shared 1.4% to the total variation was mainly contributed by 1-Hexadecanol, 1-hydroxylycopene, 2,6,6-Trimethylcyclohex-2-en-1-one, 3-Palmitoyl-sn-glycerol, 6-Octadecenoic acid, Ascorbic acid, Cholest-5-ene, Clionasterol, cis-Vaccenic acid, Dihydrobrassicasterol, Geranyl-geranyl-PP, Heptadecanoic acid, Isofucosterol 3-O-[6-O-(9,12-Octadecadienoyl)-b-D-glucopyranoside], Lignocerane, Oleic acid, Palmitic acid, Palmitoyl chloride, Paullinic acid, petroselinate and trans-12-Octadecenoic acid.

### Screening of differential metabolites

**Fig. 5:**
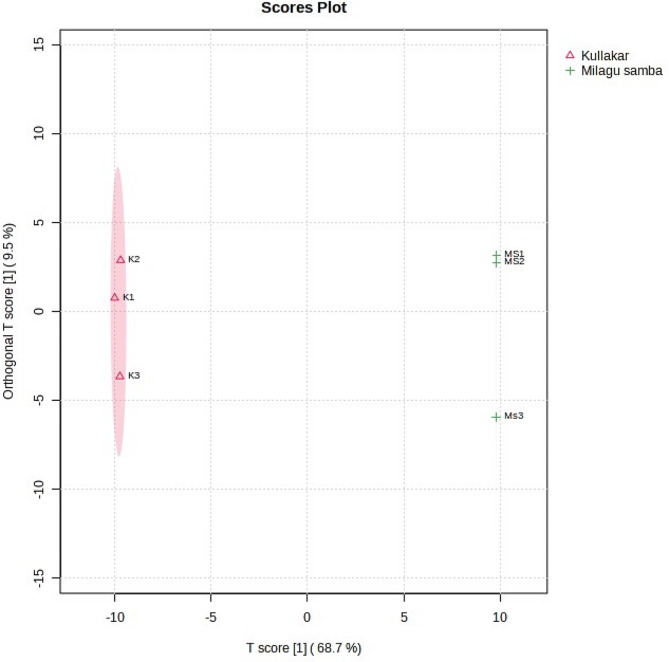
Score plot of OPLS-DA for differential metabolites analysis of pigmented rice compared to non-pigmented rice

An OPLS-DA model was constructed to precisely recognize the distinct metabolites among the 2 rice varieties. The values of R^2^Y and Q^2^ were adjacent to 1 (R^2^X = 0.687, R^2^Y = 0.999, Q^2^ = 0.977), that validates the reliability and stability of OPLS-DA model for identifying the differential metabolites between the sample groups (Fig. S4, Table S4). The score graph of OPLS-DA (Fig. 5) markedly separated the Kullakar from Milagu samba, indicating the ascertain variation in the metabolite phenotypes of pigmented and non-pigmented rice varieties. The score plot of OPLS-DA model (Fig. 5) efficiently discriminated the metabolite distribution within and between the sample groups and the organisation of samples were exactly matched the PCA score plot (Fig. 4). The PLS-DA showed similar variance like PCA, and showed 68.7% and 9.5% variance along latent variables 1 and latent variables 2, respectively (Fig. 5).

**Fig. 6:**
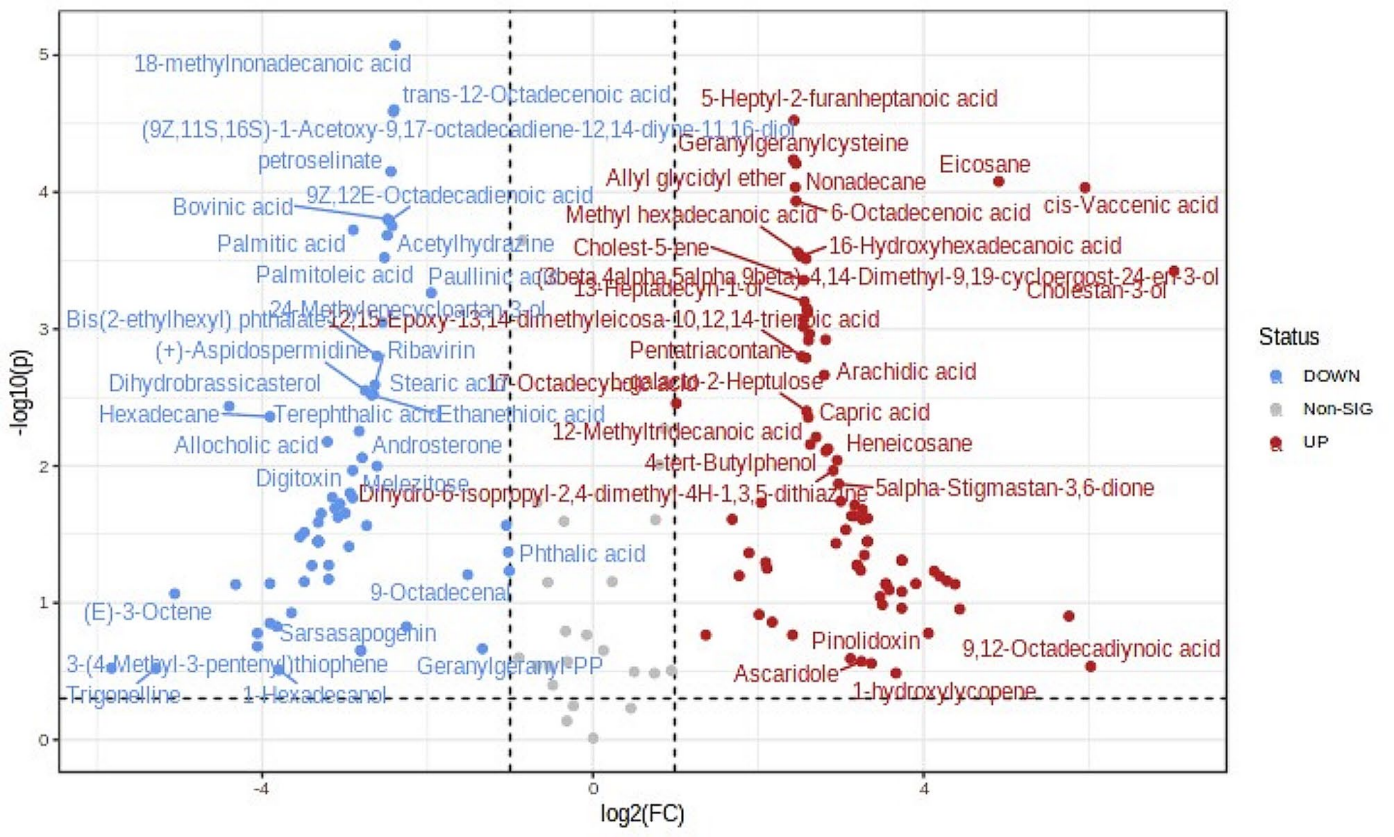
The volcano plot diagram depicts the expression levels of differential metabolites (with a fold change greater than 2) between pigmented and non-pigmented rice varieties

Differential metabolites were chosen between the 2 rice groups according to the OPLS-DA model (Fig. 5), variable importance in projection (VIP) ≥ 1 and fold change (FC) ≥ 2 or FC ≤ 0.5. The screened 144 metabolites (80 up regulated and 64 down regulated) are visually depicted through volcano plots (Fig. 6) (Table S5) that were significantly different among Kullakar and Milagu Samba.

### Variable important projection (VIP)

**Fig. 7:**
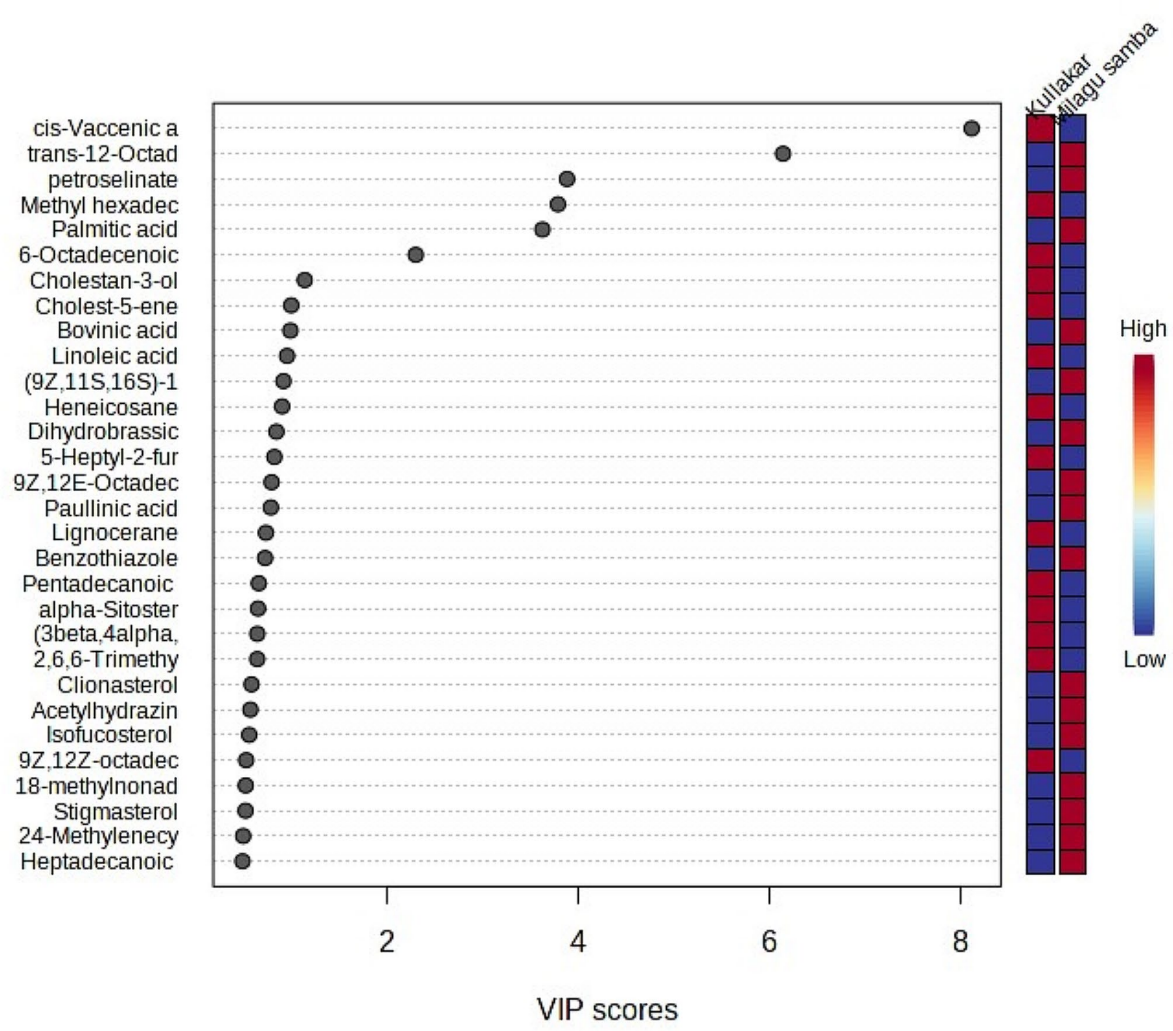
VIP scores for the selected differential metabolites

The PLS-DA model was used to calculate each metabolite’s Variable Importance in the Projection (VIP) ratings. The most significant biomarkers were found to be metabolites with a VIP score higher than 1.0. These particular metabolites have the ability to distinguish between pigmented and non-pigmented rice samples. The top 30 metabolite features having VIP value of more than one was identified from the pigmented and non-pigmented rice (Fig. 7). Most discriminated list of metabolites included Stearic acid, Paullinic acid, Cholestan-3-ol, Linoleic acid, Dihydrobrassicosterol, Lignocerane, Palmitic acid, Bovinic acid, Palmitoleic acid, Ribavirin and petroselinate, alpha-Sitosterol and Stigmasterol.

### Correlation network analysis

**Fig. 8:**
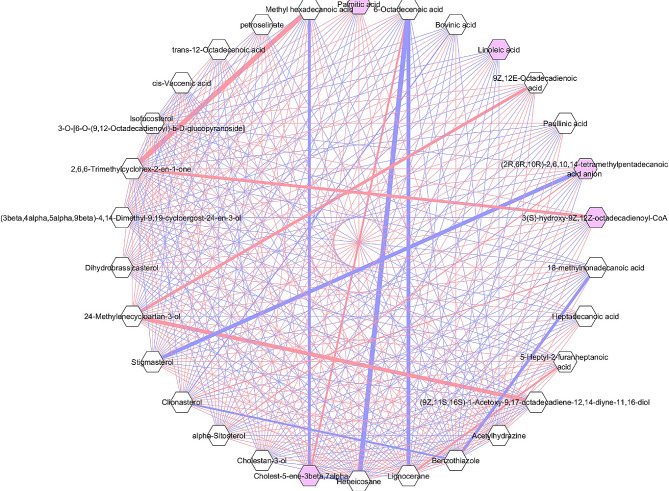
The correlation network among the VIP metabolites is visualized. Positive correlations are represented by the orange color, while negative correlations are depicted in violet. The size of the network lines reflects the strength of the associations between the metabolites

To further elucidate the importance and association of identified top 30 metabolite features correlation network was constructed using a regularization technique called the DSPC was created to handle high-dimensional metabolomics data derived from mass spectrometry [25] in Cytoscape software. A total of 30 metabolites were identified as candidate biomarkers based on OPLS-DA analysis through VIP Score among pigmented and non-pigmented varieties. A visualization of the correlation networks is presented in Fig. 8. All these 30 high influential metabolites were belonged to fatty acids, steroids, alkanes and organooxygen compounds. High positive correlation among the fatty acids were found between (9*Z*,11 S,16 S)-1-Acetoxy-9,17-octadecadiene-12,14-diyne-11,16-diol and 9Z,12*E*-Octadecadienoic acid. An organooxygen metabolite 2,6,6-Trimethylcyclohex-2-en-1-one was positively correlated with 9*Z*,12*Z*-octadecadienoyl-CoA and Methyl hexadecanoic acid. Among the steroids 24-Methylenecycloartan-3-ol was positively correlated with the fatty acid compounds like (9*Z*,11 S,16 S)-1-Acetoxy-9,17-octadecadiene-12,14-diyne-11,16-diol and 9*Z*,12*E*-Octadecadienoic acid. Alkanes namely Lignocerane and Heneicosane are having high negative correlation with 6 Octadecenoic acid. There is no negative correlation found among the fatty acids. Steroids have strong negative correlation with fatty acids.

### Pathway enrichment analysis

**Fig. 9:**
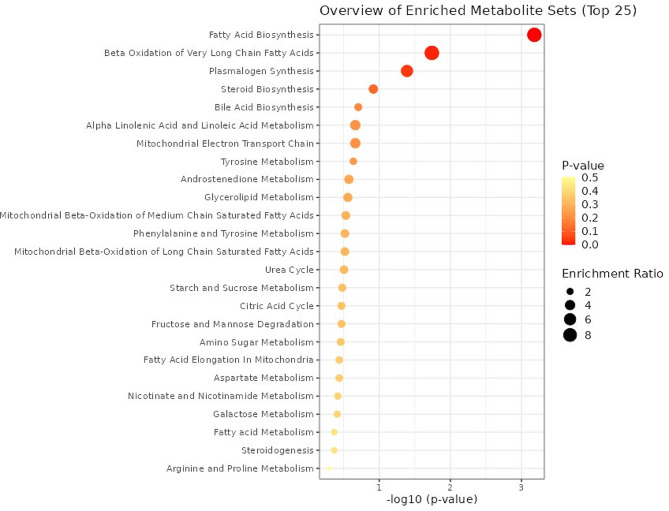
The KEGG pathway analysis was performed on distinct metabolites for pigmented and non-pigmented rice. In the visual representation, each bubble represents a metabolic pathway, with the horizontal axis indicating the extent of associated factors (larger bubbles indicating more significant impacts). The colour of the bubbles corresponds to the p-value obtained from the enrichment analysis, with lighter colors indicating lower levels of enrichment

Enrichment analysis was conducted with the purpose of revealing the vital biological role, which can greatly contribute to our understanding of the primary molecular function. The differential metabolites (144) identified through the Volcano analysis and Fold change were subjected to enrichment analysis based on the Overall representative analysis and p-values of the biological pathways that involved 27 metabolic pathways (Fig. 9; Table S6). Pathways namely Fatty Acid Biosynthesis, Beta Oxidation of Very Long Chain Fatty Acids and Plasmalogen Synthesis were enriched significantly (p-value < 0.05) in the Kullakar and Milagu Samba comparison.

## Discussion

Rice holds great significance both in terms of human impact and economic value on a global scale. Enhancing grain yield and disease resistance are the main objectives when it comes to improving crop genetics [26]. Additionally, the focus on enhancing the nutritional quality of rice has gained considerable attention in recent years [27]. Wild rice and landraces, with their diverse genetic makeup, have become crucial resources for advancing rice genetics and developing new cultivars [28]. Landraces have garnered attention for their ability to adapt to local environments, tolerate abiotic stress, and possess specific metabolic components [29]. Through ongoing domestication and improvement processes, landraces have exhibited distinct characteristics compared to cultivated rice [30].

Pigmented rice is increasingly popular due to its bioactive compounds like phenols, flavonoids, nutritional benefits, minerals, vitamins and plant sterols [11, 31, 32] amidst the COVID pandemic. Untargeted metabolomic approach was used to produce a comprehensive report on metabolomic profile of pigmented and non-pigmented rice. The study identified 168 metabolites comprised of fatty acids, sugars, steroids and benzene com-pounds which has significant role in nutritional as well as the pharmacological sectors (Table 2 & Table S1).

**Table 2:** Biological significance of metabolites identified from the study

S.No.	Important metabolites	Biological significance	References
Fatty acids			
1.	Palmitic acid	Increases the risk of cardiovascular diseases	[50]
2.	Elaidic acid	Anticancer	[51]
3.	Oleic acid	Reduces cardiovascular risk by reducing blood cholesterol	[52]
4.	Stearic acid	Reduces human breast cancer	[53]
5.	Undecanoic & Capric acid	Immune function	[54]
6.	Arachidic acid	Important for normal health	[55]
7.	Myristoleic acid	Post-translational protein changes and mechanisms that control important metabolic processes in the human body	[56]
8.	Behenic acid	*Promote the cholesterol levels in humans*	[57]
9.	cis-Vaccenic acid	*Reducing the incidence of heart disease, cancer, and obesity*	[58]
10.	Paullinic acid	Remarkable antioxidant properties and serves as a natural preservative	[59]
11.	alpha-Linolenic acid	Reduction of blood pressure, blood triglycerides, inflammation and the incidence of cardiovascular disease	[60]
12.	Linoleic acid	*Anti-inflammatory, acne reductive, skin-lightening and moisture retentive properties*	[61]
**Phenylpropanes**			
13.	2,4-Di-*tert*-butyl phenol	Antioxidant Antifungal	[62]
**Terpenoids**			
14.	Squalene	Reduces wrinkles and decreases UV-induced DNA damage in human skin	[63]
15.	gamma-tocopherol	Cardiovascular performance	[64]
16.	Hydroxy lycopene	Anticancer, Antidiabetic, Cardioprotective, Antioxidative, Anti-Inflammatory, Hepatoprotective, Neuroprotective	[65]
17.	alpha-Carotene	Antioxidant and possibly anti-carcinogenic properties, and enhance immune function	[66]
**Alkanes**			
18.	Tetradecane hexadecane and pentadecane	Antifungal and antibacterial effects	[67]
**Sterols**			
19.	Campesterol; Brassicasterol; Sitosterol; Stigmasterol	Reduce blood cholesterol levels: total cholesterol and LDL cholesterol & Inhibit cholesterol absorption	[68]
**Phenols**			
20.	-Methoxy-4-vinylphenol	Antimicrobial, antitumor, and mitigating inflammatory issues	[69]

Significant differences were observed for the total levels of covered fatty alcohols be-tween pigmented and non-pigmented rice. Moreover, pigmented rice had lower fatty acid levels than non-pigmented rice as demonstrated by [33]. Besides, both saturated and un-saturated fatty acids were also identified in both types of rice. Markedly, the pigmented rice exhibited a comparatively higher abundance of monounsaturated fatty acids (MUFAs) and polyunsaturated fatty acids (PUFAs), suggesting its efficacy in reducing cholesterol levels by raising high density lipoprotein (HDL) [34] and potentially enhancing consumer preference. Edible oils are commonly relied upon as a source of PUFAs in the diet, nevertheless the presence of polyunsaturated fatty acids (PUFAs) in traditional rice offers a valuable and cost-effective alternative for individuals who may find it challenging to afford high-value edible oils. This not only addresses nutritional needs but also contributes to dietary diversity, emphasizing the significance of locally available and affordable food sources (traditional varieties) and helps to achieve SDG 2. Moreover, the fatty acid composition of rice is closely tied to its palatability. Studies [35] report that rice cultivars with higher fatty acid content generally exhibit superior eating quality.

Differences in the composition and contents of phenolic acids among various rice varieties have been observed, potentially contributing to their bioactivities. Specifically, this study found that Kullakar had a relatively higher content of 2-Methoxy-4-vinylphenol compared to Milagu Samba. Consequently, variations in the antioxidant activities of these two varieties may be associated with discrepancies in phenolic compound levels as reported in the previous finding [11]. These findings suggest that consumer preferences during the pandemic situation could be influenced by such differences.

Vitamins and phytosterols, including campesterol, β-sitosterol, cholesterol, and stigmasterol, Dihydrobrassicasterol exhibited higher abundance in Kullakar compared to non-pigmented Milagu Samba agreeing the findings of Kim et al. [36]. Notably, these metabolites played a pivotal role in distinguishing Kullakar from Milagu Samba samples in both PCA and OPLS-DA, forming a distinct cluster separate from other metabolites. Nevertheless, levels of plant sterols are exemplary found in both types specifies the need for its consumption irrespective of pigmentation.

Content of terpenoids were higher in Kullakar except for Geranylgeranyl-PP, plays a crucial role in treating a diverse range of diseases. Numerous in vitro and in vivo studies have explored its potential applications as anticancer agents, antimicrobial agents, anti-inflammatory agents, antioxidants, antiallergic agents, neuroprotective agents, anti-aggregators, anti-coagulants, sedatives and analgesics. These therapeutic effects are attributed to the activities of monoterpenes, sesquiterpenes, diterpenes, triterpenes, tetraterpenes and glycoside compounds as outlined in the previous research [37].

In the present study, it was found that α-Tocopherol was the most prevalent form of the tocopherols analyzed. This finding is consistent with previous targeted analyses conducted on both coloured and non-coloured rice [38, 39]. According to a previous study [40], α-tocopherol levels are highest in Black rice. However, in the present study, pigmented rice contained less β-tocopherol than non-pigmented white rice.

Among the varieties studied, there were significant variations in the levels of α-tocopherol, resulting in a wide range for this compound. It is worth noting that Milagu Samba exhibited particular benzothiazoles, carbothioic S-acids, and carboxylic acid derivatives not observed in other varieties.

The PCA analysis clearly separated the Kullakar and Milagu Samba in two different clusters (Fig. 4). The PC1 and PC2 contributed to 96.4% and 1.9% of the variance, respectively. The results demonstrated that PC1 separated pigmented (Kullakar) and non-pigmented rice (Milagu Samba), indicating that genetic variation strongly influenced the metabolite profiles of pigmented and non-pigmented rice varieties, align with the previous finding [41]. The present study revealed a distinct separation based on the colour of rice is in accordance with the earlier research [39]. From the study, it is clearly evident that the due to the colour discrimination between the 2 varieties, lycopene and carotene were specific only to pigmented variety.

Furthermore, OPLS-DA was used to find potentially significant biomarker metabolites that are connected to the pigmentation of rice. It is a supervised technique, primarily utilized for sample classification and biomarker metabolite selection [42]. The interaction between two rice groups and variable compounds was established using an effective statistical tool called OPLS-DA (43). R^2^X and R^2^Y represents the percentage of the fitting equation explanations for X and Y matrices, respectively, while Q^2^ shows the fitting equations predictive ability. The coefficients of R^2^ and Q^2^ surpassing 0.5 and closer to 1.0 can be considered as accurate findings as suggested by [44]. In the present investigation (Fig. S4), established predictive model fitted the differential compounds between the pigmented and non-pigmented traditional rice varieties with predictive ability Q^2^ (cum) = 0.977, goodness-of-fit parameter R^2^X (cum) = 0.687 and variance explanatory ability R^2^Y (cum) = 0.999. The OPLS-DA score plot (Fig. 5) indicated the clear separation of these 2 varieties which corroborates with the previous findings [11].

The screened 144 metabolites are visually depicted through volcano plots (Fig. 6) (Table S5) that were significantly different among Kullakar and Milagu Samba. Eighty compounds were up regulated in Kullakar over Milagu Samba that includes Glycerolipids, Lactones, Oxocins, Phenol esters, Phenols and Thiols. The down regulated (64 compounds) metabolites comprised of Acyl halides, Aspidospermatan-type alkaloids, Benzothiazoles, Carbothioic S-acids, Carboxylic acids and derivatives, Heteroaromatic compounds, Naphthalenes, Pyridines and derivatives and Triazole ribonucleosides. Findings of this clearly indicates the discernible variations in metabolite composition of pigmented and non-pigmented rice.

The score plot of OPLS-DA model (Fig. 5) efficiently discriminated the metabolite distribution within and between the sample groups and the organisation of samples were exactly matched the PCA score plot (Fig. 4). The PLS-DA showed similar variance like PCA, and showed 68.7% and 9.5% variance along latent variables 1 and latent variables 2, respectively (Fig. 5). Same results were also reported by [45, 46].

The differential metabolites identified through OPLS-DA model was 144 (64 down regulated and 85 up regulated) and these significant differences observed between Kullakar and Milagu Samba amounted to 59 per cent. Hence, these significant values validate the notion that the influence of colour variations among rice genotypes on their metabolite profiles extend beyond environmental factors and confirm the findings of previous investigation [39].

Biomarker metabolites with a larger influence on the separation of pigmentation were chosen based on their VIP values (VIP > 1.0) in order to decrease the overall number of metabolites discovered. According to the OPLS-DA score plot, these biomarker metabolites were in charge of the clustering and separation of the pigmentation. The top 30 metabolite features having VIP value of more than one was identified from the pigmented and non-pigmented rice (Fig. 7) was aligns with the previous study [47]. Fatty acids prove to be good biomarkers for differentiating two rice groups. Most discriminated list of metabolites included Stearic acid, Paullinic acid, Cholestan-3-ol, Linoleic acid, Dihydrobras sicosterol, Lignocerane, Palmitic acid, Bovinic acid, Palmitoleic acid, Ribavirin and petroselinate, alpha-Sitosterol and Stigmasterol.

Two alkanes that show a notable negative connection with 6-Octadecenoic acid are lignocerane and heniceicosane. This implies a novel link where 6-Octadecenoic acid levels are inversely correlated with the presence or concentration of alkanes in the context of lignocerane and henocersane. Interestingly, though, there were no unfavourable associations found between the different fatty acids, suggests that each member of this class of chemicals exhibits unique and autonomous behavior.

The Kyoto Encyclopedia of Genes and Genomes (KEGG) database holds a prominent and esteemed position as the foremost public pathway database. It is highly regarded for its exceptional utility in conducting research on signal transduction pathways and the accumulation of metabolites [48]. In our study, we took great care to meticulously annotate and enhance the differential metabolites for each comparison group. These metabolites were then systematically organized into specific KEGG pathways, allowing for a more comprehensive understanding of their roles and functions. The purpose of this enrichment analysis is to identify crucial biological pathways that impact specific biological processes, thus revealing the fundamental molecular mechanisms that underlie these processes (Fig. 9). The differential metabolites (144) were subjected to enrichment analysis and identified 27 metabolic pathways (Fig. 9; Table S6). Pathways namely Fatty Acid Biosynthesis, Beta Oxidation of Very Long Chain Fatty Acids and Plasmalogen Synthesis were enriched significantly (p-value < 0.05) in the Kullakar and Milagu Samba comparison. This indicates the significant difference in the concentration of MUFAs and other differential metabolites. The red colour bubble of this pathway indicates that it has high confidence (low p values) and less chance of error. On another side, Alpha Linolenic Acid and Linoleic Acid Metabolic pathway exhibited higher bubble size but low enrichment factor, accompanied by low p values, indicating the less chance of error. Nearly 6 numbers of metabolites have been involved in this pathway.

**Fig. 10:**
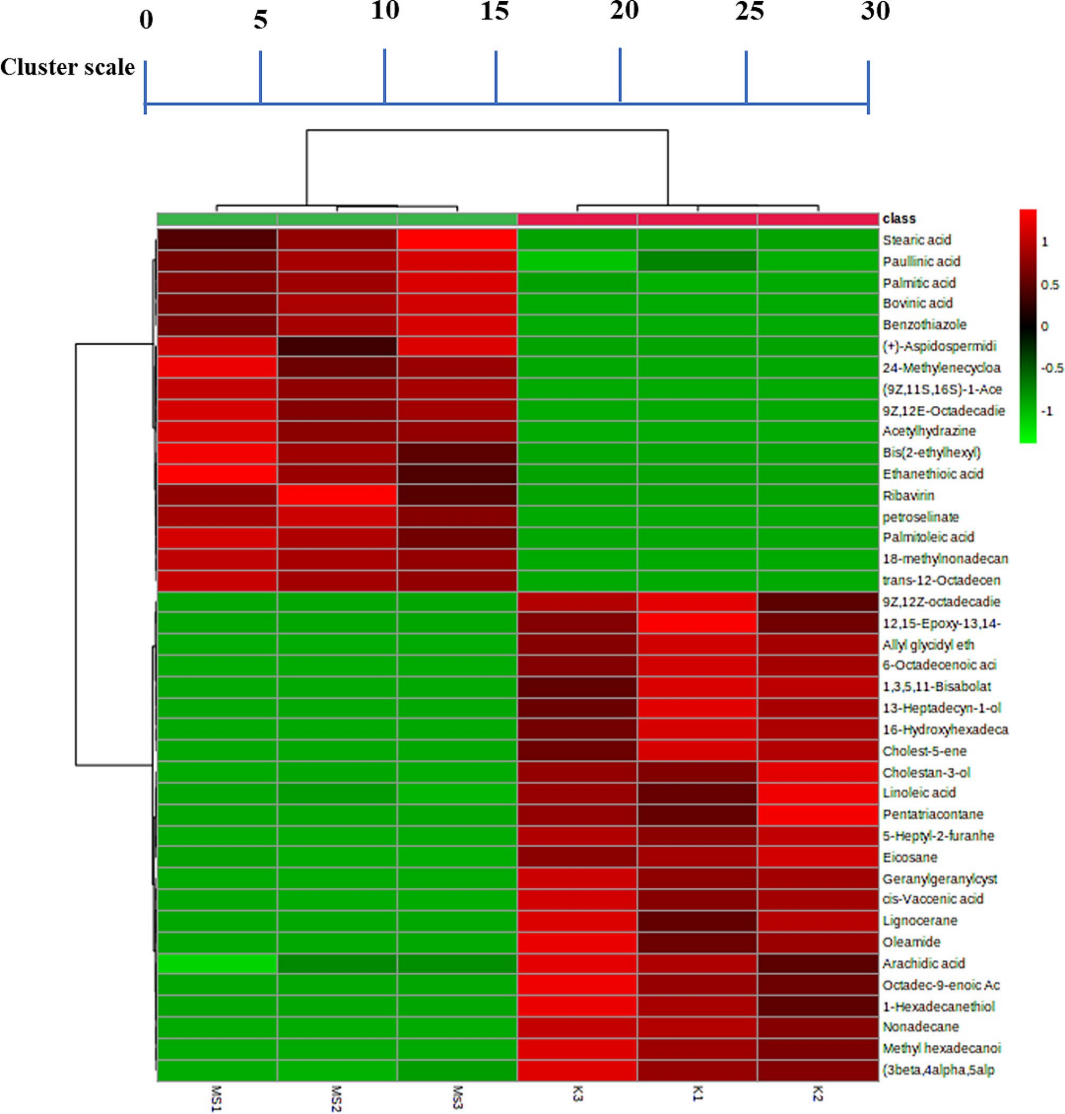
Heat map analysis showing the abundance of OPLS-DA based differential metabolites between the grains of Kullakar and Milagu Samba. Cells represented in red indicate a higher relative abundance of the metabolites in the rice samples, while green designate lower concentration. The cluster was constructed based on the fold-change heat-map, utilizing similarity measured by Euclidean distance and linkage rule by Ward’s method. K1-3 indicates Kullakar and MS1-3 denotes Milagu Samba

The heatmap presented top 40 differential metabolites are belonging to the biological classes of amino acids, sugars, sterols and alkanes. Metabolites are visually represented in a colour gradient from green to red, indicating their content in decreasing order. Cells rep-resented in red indicate a higher relative abundance of the metabolites in the rice samples, while green designate lower concentration. Interestingly, over 50% of the highlighted metabolites are found in samples with high relative abundances. The results of heatmap associated with the data resultant from PCA, confirming the evenness of the results. Ac-cording to the present study heatmap (Fig. 9), Stearic acid, Paullinic acid, Palmitic acid, Bovinic acid, Palmitoleic acid, Ribavirin and (+)-Aspidospermidine had lower relative abundance among the Kullakar replications compared to Milagu Samba. These variations revealed that the metabolite composition is primarily influenced by the pigmentation of the variety. Present study confirms the findings of [49] (Fig. 10).

In order to meet Sustainable Development Goal 2 (SDG 2) of the UN, it is imperative to cultivate nutritious traditional rice varieties. Due to their rich and varied nutrient pro-files, these cultivars greatly improve nutrition and global food security. Traditional rice varieties are strongly aligned with SDG 2’s commitment to ensuring that everyone has ac-cess to safe and nutritious food, as they have the potential to prevent malnutrition and correct specific nutrient deficiencies. Their production also promotes resilient agriculture, climate change adaptation, and the preservation of agricultural biodiversity. In addition to empowering communities and protecting cultural heritage, states can support SDG 2’s larger goals of fostering a more resilient, diverse, and sustainable global food system by encouraging the production and consumption of these varieties.

## Conclusions

This study presents a comprehensive examination of the range of metabolites found in Kullakar and Milagu samba, which has not been done before. By utilizing relevant sta-tistical methods, researchers were able to analyze and evaluate the variations in metabolism between pigmented and non-pigmented rice. With the progression of the social economy and improvements in living standards, the occurrence of a pandemic situation adds a complex dynamic. To fulfil the aim of achieving Sustainable Development Goal 2 (SDG 2) for zero hunger and proper nutrition, there is an escalating demand for higher quality rice among consumers. Consequently, there has been an enhanced interest in consuming traditional rice varieties. The future trajectory of seed industry development will likely prioritize the breeding of rice varieties with superior eating quality. These findings not only provide valuable insights into the metabolite composition of pigmented and non-pigmented rice grains but also lay the groundwork to expedite the breeding of rice varieties with superior functional and nutritional significance.

### Electronic supplementary material

Below is the link to the electronic supplementary material.

Supplementary Material 1

## Data Availability

The datasets generated and analyzed in the current study are available from the corresponding author on reasonable request. All data generated or analyzed during this study are included in this published article and its Supplementary information files.

## Abbreviations

SDG 2: Sustainable Development Goal - Zero Hunger
PCA: Principal Component Analysis
OPLS-DA: Orthogonal Partial Least Square Discrimination Analysis
KEGG: Kyoto Encyclopedia of Genes and Genomes
GC-MS/MS: Gas Chromatography-Mass Spectrometry
PVDF: Polyvinylidene fluoride
RPM: Rotation Per Minute
HMDB: Human Metabolome Database
NIST: National Institute of Standards and Technology
MSEA: Metabolite Sets Enrichment Analysis
HPLC: High-performance liquid chromatography
VIP: variable importance in the projection
DSPC: Debiased Sparse Partial Correlation algorithm
FC: Fold Change
MUFAs: Monounsaturated Fatty Acids
PUFAs: Polyunsaturated Fatty Acids
HDL: High Density Lipoprotein
