# Supplementary material for: Unravelling the metabolomic diversity of pigmented and non-pigmented traditional rice from Tamil Nadu, India
Source: BMC Plant Biol. 2024 May 15;24:402. doi: 10.1186/s12870-024-05123-3 (PMC11095017; doi:10.1186/s12870-024-05123-3)
Supplement: Supplementary file 1 — Supplementary Material 1 [file 12870_2024_5123_MOESM1_ESM.docx]

Unravelling the metabolomic diversity of pigmented and non-pigmented traditional rice from Tamil Nadu, India

Subramanian Venkatesan^1^, Dhandayuthapani Udhaya Nandhini^2^, Kandasamy Senthilraja^1*^, Sivaprakasam Jidhu Vaishnavi^3^, Balasubramaniam Prabha^4^, Shanmugam Mohan Kumar^5^, Nagappan Sriram^1^, Elangovan Subramanian^6^, Subramani Umesh Kanna^7^, Eagan Somasundaram^8^, Vellingiri Geethalakshmi^5^ and Muthurajan Raveendran^1^*

^1^ Directorate of Research, Tamil Nadu Agricultural University, Coimbatore-641 003, Tamil Nadu, India

^2^ Centre of Excellence in sustaining Soil Health, Anbil Dharmalingam Agricultural College & Research Institute, Trichy-620 027, Tamil Nadu, India

^3^ Amrita School of Agricultural Sciences, Coimbatore 642 109, Tamil Nadu, India

^4^ Department of Renewable Energy Engineering, Tamil Nadu Agricultural University, Coimbatore 641 003, Tamil Nadu, India

^5^ Agro-Climatic Research Centre, Tamil Nadu Agricultural University, Coimbatore 641 003, Tamil Nadu, India

^6^ Krishi Vigyan Kendra, Madurai, Tamil Nadu – 625 104

^7^ Directorate of Planning and Monitoring, Tamil Nadu Agricultural University, Coimbatore 641 003, Tamil Nadu, India

^8^ Agribusiness Development, Tamil Nadu Agricultural University, Coimbatore-641 003. Tamil Nadu, India

* Corresponding author E-mail: [raveendrantnau@gmail.com](mailto:raveendrantnau@gmail.com); senthilrajaens@gmail.com

**Figure S1. Chromatogram of Kullakar (Pigmented traditional rice)**

**Figure S2. Chromatogram of Milagu Samba (Non-pigmented traditional rice)**


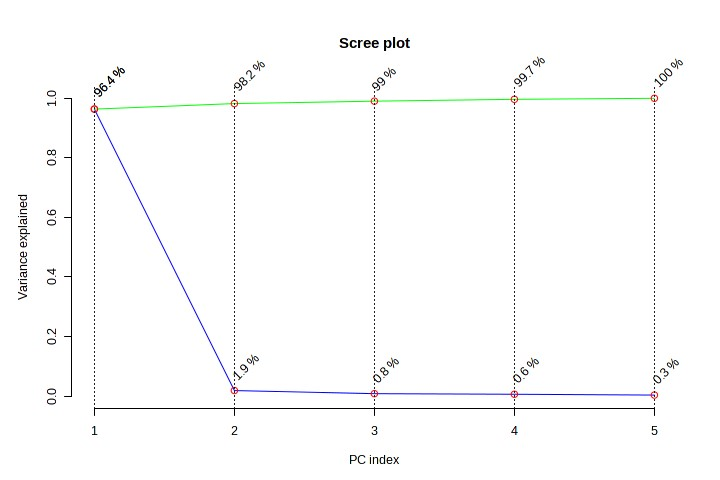


**Figure S3. Scree plot of PCA.**


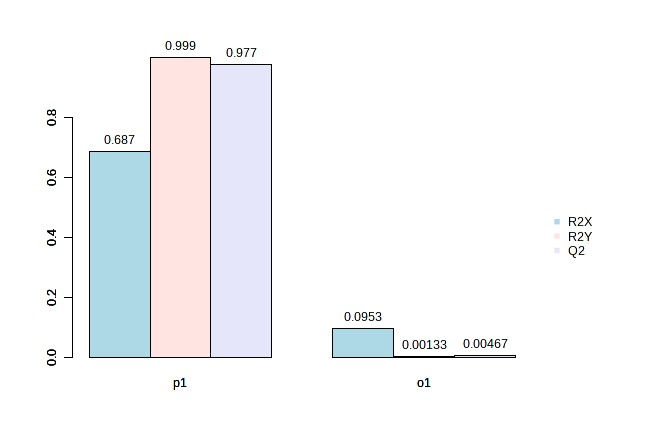


**Fig. S4. OPLS-DA permutation plot. Kullkar vs Milagu Samba.**

**Table S1. Identified 167 metabolites based on their composition**

| **S.No** | **RT** | **Name** | **Concentration of metabolites (%)** | | **Class** |
| --- | --- | --- | --- | --- | --- |
|  |  |  | **Kullakar** | **Milagu Samba** |  |
| 1 | 18.73 | 4-Ethyl-2-methoxyphenol |  | 0.06 | Phenols |
| 2 | 18.14 | 4-{3-[3-(3,5-dihydroxyphenyl)-6-hydroxy-2-(4-hydroxyphenyl)-2,3-dihydro-1-benzofuran-4-yl]-5-{2-[6-hydroxy-3-(3-hydroxyphenyl)-2-phenyl-2,3-dihydro-1-benzofuran-4-yl]ethenyl}-2,3-dihydro-1-benzofuran-2-yl}benzene-1,2-diol | 0.08 |  | 2-arylbenzofuran flavonoids |
| 3 | 22.96 | Palmitoyl chloride | 0.49 | 0.92 | Acyl halides |
| 4 | 18.39 | (+)-Aspidospermidine | - | 0.03 | Aspidospermatan-type alkaloids |
| 5 | 18.68 | Dihydro-6-isopropyl-2,4-dimethyl-4H-1,3,5-dithiazine | 0.03 |  | Azacyclic compounds |
| 6 | 18.73 | 1-Phenylethanol |  | 0.06 | Benzene and substituted derivatives |
| 7 | 20.19 | 2,4-Di-tert-butylphenol | 0.40 |  | Benzene and substituted derivatives |
| 8 | 20.19 | 2,6-Ditert-butyl-4-[(3,5-ditert-butyl-4-hydroxyphenyl)sulfanylmethylsulfanyl]phenol | 0.40 |  | Benzene and substituted derivatives |
| 9 | 18.68 | Phenylacetaldehyde | 0.03 |  | Benzene and substituted derivatives |
| 10 | 21.66 | Phthalic acid | 0.66 | 0.95 | Benzene and substituted derivatives |
| 11 | 25.86 | Terephthalic acid |  | 1.12 | Benzene and substituted derivatives |
| 12 | 18.73 | 1,4-Dimethoxybenzene | 0.04 | - | Benzene and substituted derivatives |
| 13 | 16.76 | 1-hydroperoxy-8-carboxyoctyl-3,4-epoxynon-(2E)-enyl-ether | - | 0.01 | Benzene and substituted derivatives |
| 14 | 19.02 | 4-tert-Butylphenol | 0.24 |  | Benzene and substituted derivatives |
| 15 | 18.14 | Benzene | 0.08 | 0.06 | Benzene and substituted derivatives |
| 16 | 24.45 | Bis(2-ethylhexyl) phthalate |  | 0.95 | Benzene and substituted derivatives |
| 17 | 18.84 | 3,6-Dimethyl-2(3H)-benzofuranone | 0.06 |  | Benzofurans |
| 18 | 22.03 | Benzothiazole |  | 2.39 | Benzothiazoles |
| 19 | 23.90 | Ethanethioic acid |  | 0.68 | Carbothioic S-acids |
| 20 | 22.03 | Acetylhydrazine |  | 2.39 | Carboxylic acids and derivatives |
| 21 | 23.90 | Fumaric acid |  | 0.68 | Carboxylic acids and derivatives |
| 22 | 17.70 | 2-(2-Amino-1H-benzo[d]imidazol-1-yl)-N-benzyl-8-methoxyquinazolin-4-amine | 0.36 |  | Diazanaphthalenes |
| 23 | 21.94 | Ascorbic acid | 13.63 | 11.49 | Dihydrofurans |
| 24 | 18.94 | Ascaridole | 0.00 |  | Dioxanes |
| 25 | 24.00 | Allyl glycidyl ether | 1.17 |  | Epoxides |
| 26 | 23.75 | (9Z,11S,16S)-1-Acetoxy-9,17-octadecadiene-12,14-diyne-11,16-diol |  | 3.66 | Fatty Acyls |
| 27 | 21.06 | 12-Methyltridecanoic acid | 0.17 | - | Fatty Acyls |
| 28 | 23.26 | 16-Hydroxyhexadecanoic acid | 1.34 | - | Fatty Acyls |
| 29 | 24.85 | 20-methyldocosanoic acid |  | 0.41 | Fatty Acyls |
| 30 | 30.42 | 5,8,11-Eicosatriynoic acid | 0.47 |  | Fatty Acyls |
| 31 | 22.42 | 6-Octadecenoic acid | 9.38 |  | Fatty Acyls |
| 32 | 24.00 | alpha-Linolenic acid | 1.17 | 1.91 | Fatty Acyls |
| 33 | 25.60 | Arachidic acid | 0.57 | 0.01 | Fatty Acyls |
| 34 | 19.33 | Capric acid | 0.25 |  | Fatty Acyls |
| 35 | 20.38 | Dodecanoic acid | 1.04 | 0.32 | Fatty Acyls |
| 36 | 25.60 | Heneicosanoic acid | 0.57 | 0.53 | Fatty Acyls |
| 37 | 21.06 | Methyl tetradecanoate | 0.17 | 0.37 | Fatty Acyls |
| 38 | 20.38 | Myristic acid | 1.04 | 0.44 | Fatty Acyls |
| 39 | 19.49 | Myristoleic acid | 0.02 | 0.02 | Fatty Acyls |
| 40 | 17.87 | Palmitelaidic acid | 0.02 |  | Fatty Acyls |
| 41 | 20.75 | Panaxydol |  | 0.02 | Fatty Acyls |
| 42 | 20.63 | Stearaldehyde | 0.03 |  | Fatty Acyls |
| 43 | 25.60 | Tetracosanoic acid | 0.57 | 0.53 | Fatty Acyls |
| 44 | 20.63 | Tetradec-2-enal | 0.03 |  | Fatty Acyls |
| 45 | 22.57 | trans-12-Octadecenoic acid |  | 24.56 | Fatty Acyls |
| 46 | 15.74 | Tridecanedioyl-CoA | 0.00 |  | Fatty Acyls |
| 47 | 20.20 | Undecanoic acid |  | 0.22 | Fatty Acyls |
| 48 | 17.33 | (2E)-2-dodecen-1-ol | 0.10 | - | Fatty Acyls |
| 49 | 21.59 | (E,E)-11,13-Octadecadien-9-ynoic acid | 0.15 | - | Fatty Acyls |
| 50 | 23.48 | (E,E)-11,13-Octadecadien-9-ynoic acid | - | 0.35 | Fatty Acyls |
| 51 | 22.57 | (Z)-13-Octadecenoic acid | 22.58 | 24.56 | Fatty Acyls |
| 52 | 18.60 | 10Z-Nonadecenoic acid | - | 0.02 | Fatty Acyls |
| 53 | 22.96 | 12,15-Epoxy-13,14-dimethyleicosa-10,12,14-trienoic acid | 0.49 | - | Fatty Acyls |
| 54 | 22.79 | 13-Heptadecyn-1-ol | 1.16 | - | Fatty Acyls |
| 55 | 4.30 | 13-Heptadecyn-1-ol | - | 0.03 | Fatty Acyls |
| 56 | 22.79 | 17-Octadecynoic acid | 1.16 | 0.60 | Fatty Acyls |
| 57 | 23.26 | 18-methylnonadecanoic acid | - | 2.06 | Fatty Acyls |
| 58 | 16.73 | 1-Hexadecanol | 0.00 | 0.07 | Fatty Acyls |
| 59 | 20.89 | 1-Undecanol |  | 0.07 | Fatty Acyls |
| 60 | 16.03 | 2-Nonen-1-ol | 0.10 | 0.12 | Fatty Acyls |
| 61 | 30.42 | 5,8,11-Eicosatriynoic acid |  | 0.11 | Fatty Acyls |
| 62 | 22.72 | 5-Heptyl-2-furanheptanoic acid | 2.84 |  | Fatty Acyls |
| 63 | 19.76 | 9,12-Octadecadiynoic acid | 0.01 |  | Fatty Acyls |
| 64 | 17.06 | 9-Octadecenal | 0.00 | 0.03 | Fatty Acyls |
| 65 | 22.72 | 9Z,12E-Octadecadienoic acid |  | 3.27 | Fatty Acyls |
| 66 | 23.75 | 9Z,12Z-octadecadienoyl-CoA | 2.21 |  | Fatty Acyls |
| 67 | 22.72 | Bovinic acid |  | 3.27 | Fatty Acyls |
| 68 | 22.57 | cis-Vaccenic acid | 22.58 | 0.60 | Fatty Acyls |
| 69 | 23.49 | Elaidic acid | 2.47 | 1.53 | Fatty Acyls |
| 70 | 22.49 | Heptadecanoic acid | 2.27 | 3.39 | Fatty Acyls |
| 71 | 27.64 | Hexacosanoic acid | 0.23 | 0.10 | Fatty Acyls |
| 72 | 22.72 | Linoleic acid | 2.84 | 0.49 | Fatty Acyls |
| 73 | 21.94 | Methyl hexadecanoic acid | 13.63 |  | Fatty Acyls |
| 74 | 22.49 | Methyl stearate | 2.27 | 3.39 | Fatty Acyls |
| 75 | 27.02 | Oleamide | 1.08 |  | Fatty Acyls |
| 76 | 22.57 | Oleic acid | 22.58 | 24.56 | Fatty Acyls |
| 77 | 21.74 | Palmitoleic acid |  | 0.23 | Fatty Acyls |
| 78 | 27.02 | Paullinic acid | 1.08 | 2.88 | Fatty Acyls |
| 79 | 21.94 | Pentadecanoic acid | 13.63 | 11.49 | Fatty Acyls |
| 80 | 22.42 | petroselinate |  | 14.35 | Fatty Acyls |
| 81 | 24.23 | Stearic acid |  | 0.46 | Fatty Acyls |
| 82 | 20.38 | Tridecanoic acid | 1.04 | 0.44 | Fatty Acyls |
| 83 | 19.33 | Undecanoic acid | 0.25 |  | Fatty Acyls |
| 84 | 24.23 | Behenic acid |  | 0.46 | Fatty Acyls |
| 85 | 18.55 | cis-4-Decenol | 0.28 |  | Fatty Acyls |
| 86 | 22.79 | Octadec-9-enoic Acid | 1.16 |  | Fatty Acyls |
| 87 | 24.06 | Palmitic acid | 1.51 | 11.49 | Fatty Acyls |
| 88 | 18.00 | isosorbide | 0.15 | 0.18 | Furofurans |
| 89 | 24.19 | 3-Palmitoyl-sn-glycerol | 1.10 | 0.45 | Glycerolipids |
| 90 | 18.86 | 3-(4-Methyl-3-pentenyl)thiophene |  | 0.03 | Heteroaromatic compounds |
| 91 | 18.55 | (-)-(Z)-Tetrahydro-6-(2-pentenyl)-2H-pyran-2-one | 0.28 | - | Lactones |
| 92 | 23.75 | Isofucosterol 3-O-[6-O-(9,12-Octadecadienoyl)-b-D-glucopyranoside] | 2.21 | 3.66 | Naphthalenes |
| 93 | 18.84 | (E,E)-2,4-Decadienal | 0.06 | - | Organooxygen compounds |
| 94 | 18.55 | (Z)-2-decenal | - | 0.03 | Organooxygen compounds |
| 95 | 21.53 | 2-(Octadecyloxy)ethanol | 0.54 | 0.03 | Organooxygen compounds |
| 96 | 18.55 | 2-Decenal | 0.28 | 0.03 | Organooxygen compounds |
| 97 | 19.02 | 2'-Hydroxy-4',6'-dimethoxy-3'-methylacetophenone | 0.24 | 0.25 | Organooxygen compounds |
| 98 | 20.75 | 2-Nonadecanone | 0.02 |  | Organooxygen compounds |
| 99 | 21.53 | 2-Pentadecanone | 0.54 |  | Organooxygen compounds |
| 100 | 31.97 | 3beta-3-Lupanol |  | 0.07 | Organooxygen compounds |
| 101 | 18.40 | 4-Oxononanal | 0.14 |  | Organooxygen compounds |
| 102 | 22.26 | D-Fructose |  | 0.01 | Organooxygen compounds |
| 103 | 18.00 | L-galacto-2-Heptulose | 0.15 |  | Organooxygen compounds |
| 104 | 16.03 | Nonanal | 0.10 |  | Organooxygen compounds |
| 105 | 6.04 | Solketal | 0.01 |  | Organooxygen compounds |
| 106 | 18.14 | xi-3-Methyl-3-cyclohexen-1-ol |  | 0.14 | Organooxygen compounds |
| 107 | 19.01 | 2'-Hydroxy-5'-methylacetophenone |  | 0.25 | Organooxygen compounds |
| 108 | 18.40 | 3-Methyl-2,4-nonanedione | 0.14 |  | Organooxygen compounds |
| 109 | 18.23 | 5-Hydroxymethyl-2-furancarboxaldehyde | 0.60 | 0.95 | Organooxygen compounds |
| 110 | 29.04 | 2,6,6-Trimethylcyclohex-2-en-1-one | 2.25 |  | Organooxygen compounds |
| 111 | 18.39 | Melezitose |  | 0.03 | Organooxygen compounds |
| 112 | 17.06 | Pinolidoxin | 0.00 |  | Oxocins |
| 113 | 18.14 | Aceteugenol | 0.08 |  | Phenol esters |
| 114 | 19.02 | 2-Methoxy-4-vinylphenol | 0.24 | 0.25 | Phenols |
| 115 | 18.73 | 3-Methoxytyramine | 0.04 |  | Phenols |
| 116 | 17.33 | Bentonite | 0.10 |  | Post-transition metal organides |
| 117 | 28.59 | 1-hydroxylycopene | 0.06 | 0.03 | Prenol lipids |
| 118 | 18.94 | gamma-Ionone | 0.00 |  | Prenol lipids |
| 119 | 26.61 | Geranylgeranyl-PP | 0.18 | 0.54 | Prenol lipids |
| 120 | 29.91 | Sarsasapogenin |  | 0.00 | Prenol lipids |
| 121 | 26.61 | Squalene | 0.18 | 0.54 | Prenol lipids |
| 122 | 29.68 | Vitamin E Nicotinate |  | 0.14 | Prenol lipids |
| 123 | 18.23 | 1,3,5,11-Bisabolatetraen-10-one | 0.60 | - | Prenol lipids |
| 124 | 32.08 | alpha-Carotene | 0.00 |  | Prenol lipids |
| 125 | 29.91 | Carnosic acid |  | 0.00 | Prenol lipids |
| 126 | 29.49 | Lycophyll |  | 0.03 | Prenol lipids |
| 127 | 18.40 | Chlordecone | 0.14 |  | Prenol lipids |
| 128 | 29.68 | gamma-Tocopherol | 0.03 | 0.14 | Prenol lipids |
| 129 | 26.61 | Geranylgeranylcysteine | 0.18 |  | Prenol lipids |
| 130 | 16.90 | Methyl nicotinate |  | 0.09 | Pyridines and derivatives |
| 131 | 16.90 | Nicotinic acid |  | 0.09 | Pyridines and derivatives |
| 132 | 21.34 | 2,3,5,8-Tetramethyldecane | 0.10 |  | Saturated hydrocarbons |
| 133 | 25.34 | Eicosane | 1.37 | 0.07 | Saturated hydrocarbons |
| 134 | 15.74 | Hexadecane | 0.00 | 0.13 | Saturated hydrocarbons |
| 135 | 16.76 | Hexane |  | 0.01 | Saturated hydrocarbons |
| 136 | 25.34 | Nonadecane | 1.37 |  | Saturated hydrocarbons |
| 137 | 25.06 | N-Triacontane |  | 0.24 | Saturated hydrocarbons |
| 138 | 27.02 | Pentatriacontane | 1.08 |  | Saturated hydrocarbons |
| 139 | 21.34 | Heptadecane | 0.10 | 0.13 | Saturated hydrocarbons |
| 140 | 27.23 | Lignocerane | 2.22 |  | Saturated hydrocarbons |
| 141 | 27.23 | Octadecane |  | 0.18 | Saturated hydrocarbons |
| 142 | 20.96 | Pentadecane |  | 0.07 | Saturated hydrocarbons |
| 143 | 27.23 | Tetradecane |  | 0.18 | Saturated hydrocarbons |
| 144 | 27.23 | Heneicosane | 2.22 |  | Saturated hydrocarbons |
| 145 | 29.04 | (3beta,4alpha,5alpha,9beta)-4,14-Dimethyl-9,19-cycloergost-24-en-3-ol | 2.25 | 0.38 | Steroids and steroid derivatives |
| 146 | 25.95 | 1,25-Dihydroxyvitamin D3-26,23-lactone | 0.18 | 0.10 | Steroids and steroid derivatives |
| 147 | 19.49 | 16b-Hydroxyestradiol | 0.02 | - | Steroids and steroid derivatives |
| 148 | 19.89 | Allocholic acid | 0.04 | 0.31 | Steroids and steroid derivatives |
| 149 | 24.76 | alpha-Sitosterol | 4.09 | 2.94 | Steroids and steroid derivatives |
| 150 | 31.17 | Cholesterol | 0.56 |  | Steroids and steroid derivatives |
| 151 | 29.33 | Ergost-4-en-3-one | 0.39 |  | Steroids and steroid derivatives |
| 152 | 29.04 | 24-Methylenecycloartan-3-ol |  | 2.14 | Steroids and steroid derivatives |
| 153 | 30.79 | 5alpha-Stigmastan-3,6-dione | 0.51 |  | Steroids and steroid derivatives |
| 154 | 31.16 | Androsterone |  | 0.24 | Steroids and steroid derivatives |
| 155 | 22.35 | Campesterol | 0.39 | 0.27 | Steroids and steroid derivatives |
| 156 | 24.76 | Cholest-5-ene | 4.09 |  | Steroids and steroid derivatives |
| 157 | 30.42 | Cholesta-4,6-dien-3-one | 0.11 |  | Steroids and steroid derivatives |
| 158 | 23.19 | Cholestan-3-ol | 3.08 | 0.01 | Steroids and steroid derivatives |
| 159 | 22.35 | Dihydrobrassicasterol | 0.32 | 2.94 | Steroids and steroid derivatives |
| 160 | 26.52 | 3beta-Ergosta-5,23-dien-3-ol | 0.05 |  | Steroids and steroid derivatives |
| 161 | 24.75 | Clionasterol |  | 2.94 | Steroids and steroid derivatives |
| 162 | 20.80 | Digitoxin |  | 0.03 | Steroids and steroid derivatives |
| 163 | 23.19 | Stigmasterol | 3.08 | 4.21 | Steroids and steroid derivatives |
| 164 | 24.06 | 1-Hexadecanethiol | 1.51 |  | Thiols |
| 165 | 17.99 | Ribavirin |  | 0.18 | Triazole ribonucleosides and ribonucleotides |
| 166 | 17.33 | (6E,8E)-4,6,8-Megastigmatriene | 0.10 | - | Unsaturated hydrocarbons |
| 167 | 16.76 | (E)-3-Octene | - | 0.01 | Unsaturated hydrocarbons |

**Table S2. Venn daigram results of metabolites commonly present between pigmented and non-pigmented rice**

| **S.No** | **Traditional varieties** | **Total No. of Metabolites identified** | **Compound Name** |
| --- | --- | --- | --- |
| 1 | Kullakar Vs Milagu Samba | 49 | "9,12-Octadecadienoyl chloride, (Z,Z)-" 9-Hexadecenoic acid Glycerol 1-palmitate cis-Vaccenic acid "5,8,11-Eicosatriynoic acid, methyl ester" Ã§-Tocopherol "9,19-Cycloergost-24(28)-en-3-ol, 4,14-dimethyl-, acetate, (3Ã¡,4Ã ,5Ã )-" Squalene cis-13-Eicosenoic acid Methyl tetradecanoate "9,17-Octadecadienal, (Z)-" Oleic Acid "Benzene, 1,4-dimethoxy-2-methyl-" "1,25-Dihydroxyvitamin D3, TMS derivative" "Ergost-5-en-3-ol, acetate, (3Ã¡,24R)-" trans-Geranylgeraniol n-Hexadecanoic acid "9,12-Octadecadienoic acid, methyl ester, (E,E)-" Palmitoyl chloride "Heptadecane, 2,6,10,15-tetramethyl-" "l-(+)-Ascorbic acid 2,6-dihexadecanoate" 13-Heptadecyn-1-ol "Tetracosanoic acid, methyl ester" Ethyl iso-allocholate 4-Hydroxy-2-methylacetophenone "6-Octadecenoic acid, methyl ester, (Z)-" 17-Octadecynoic acid 2-Methoxy-4-vinylphenol "Phenol, 4-ethyl-2-methoxy-" Dodecanoic acid "2-Decenal, (E)-" "2-Decenal, (Z)-" Z-8-Methyl-9-tetradecenoic acid Pentadecanoic acid "9,12,15-Octadecatrienoic acid, 2,3-dihydroxypropyl ester, (Z,Z,Z)-" cis-13-Octadecenoic acid "Hexacosanoic acid, methyl ester" "Ethanol, 2-(9-octadecenyloxy)-, (Z)-" 5-Hydroxymethylfurfural "Heneicosanoic acid, methyl ester" "2-Nonen-1-ol, (E)-" Tridecanoic acid Ã§-Sitosterol Tetradecanoic acid "12-Methyl-E,E-2,13-octadecadien-1-ol" Rhodopin Methyl stearate "1,4:3,6-Dianhydro-Ã -d-glucopyranose" Campesterol |
| 2 | Kullakar | 65 | Nonanal "Z,Z-3,15-Octadecadien-1-ol acetate" "2(3H)-Benzofuranone, hexahydro-3-methylene-" "Heptadecanoic acid, 10-methyl-, methyl ester" Tridecanedial 4-Oxononanal Stigmasterol "Tetracosane, 11-decyl-" Cholesterol Glycidyl oleate "Nonadecane, 2-methyl-" "Carotene, 1,1',2,2'-tetrahydro-1,1'-dimethoxy-" "Benzofuran, 2,3-dihydro-" "Silane, cyclohexyldimethoxymethyl-" Methyl 16-hydroxy-hexadecanoate "8,9-Dimethylbicyclo[4.4.1]undeca-2,4,8-triene" "Decane, 2,3,5,8-tetramethyl-" "1-Hexadecanol, 2-methyl-" tert-Hexadecanethiol "Phthalic acid, isobutyl octadecyl ester" "(2E,6E,10E)-3,7,11,15-Tetramethylhexadeca-2,6,10,14-tetraen-1-yl formate" Ã¡-Tocopherol Undecanoic acid "Phenol, 4-ethenyl-, acetate" "9-Octadecenoic acid (Z)-, 2-hydroxy-1-(hydroxymethyl)ethyl ester" "5-Cholestene-3-ol, 24-methyl-" Dimethyl(isopropyl)silyloxycyclohexane 17-Pentatriacontene "2,4-Di-tert-butylphenol" "Phenol, p-tert-butyl-" "Stigmast-5-en-3-ol, oleate" "2,7-Anhydro-l-galacto-heptulofuranose" "4-Hepten-3-one, 4-methyl-" E-2-Tetradecen-1-ol "9-Octadecene, 1,1'-[1,2-ethanediylbis(oxy)]bis-, (Z,Z)-" "Tridecanoic acid, 12-methyl-, methyl ester" 5-Decanone "Z-(13,14-Epoxy)tetradec-11-en-1-ol acetate" (Z)-18-Octadec-9-enolide Ascaridole epoxide "2,4-Decadienal, (E,E)-" "Heneicosane, 11-(1-ethylpropyl)-" "2-[4-methyl-6-(2,6,6-trimethylcyclohex-1-enyl)hexa-1,3,5-trienyl]cyclohex-1-en-1-carboxaldehyde" "Phenol, 2,6-bis(1,1-dimethylethyl)-" "2H-Oxecin-2-one, 3,4,7,8,9,10-hexahydro-4-hydroxy-10-methyl-, [4S-(4R*,5E,10S*)]-" "Benzene, (ethenyloxy)-" "1,3-Dioxolane-4-methanol, 2,2-dimethyl-" 3-(1-Benzyl-1H-imidazol-2-yl)-5-methylisoxazole "Ergosta-5,22-dien-3-ol, acetate, (3Ã¡,22E)-" ï»¿cis-7-Decen-1-al "Hexadecanoic acid, 2-(octadecyloxy)ethyl ester" "2-Pentadecanone, 6,10,14-trimethyl-" Eicosane "1,5,5-Trimethyl-6-methylene-cyclohexene" "2-Dodecen-1-ol, 12-chloro-" 4-Octadecenal "2,4-Nonanedione" "13,16-Octadecadiynoic acid, methyl ester" "4-(2,2-Dimethyl-6-methylenecyclohexyl)butanal" Octadecanal "Cholesta-22,24-dien-5-ol, 4,4-dimethyl-" Methyl 14-methyl-eicosanoate "8,11-Octadecadienoic acid, methyl ester" "Benzeneacetaldehyde, Ã -ethyl-" "Decanoic acid, silver(1+) salt" "Estra-1,3,5(10)-trien-17Ã¡-ol" "Hexadecane, 1,1-bis(dodecyloxy)-" |
| 2 | Milagu Samba | 54 | Panaxydol ".psi.,.psi.-Carotene, 1,1',2,2'-tetrahydro-1,1'-dimethoxy-" "Methyl 9-cis,11-trans-octadecadienoate" "Octadecane, 3-ethyl-5-(2-ethylbutyl)-" "3,4-Dihydro-3,5,8-trimethyl-3-(4,8,12-trimethyltridecyl)-(2H)1-benzopyran-6-acetate" "D-Fructose, diethyl mercaptal, pentaacetate" Methyl 18-methylnonadecanoate cis-10-Nonadecenoic acid "Tetradecane, 2,6,10-trimethyl-" "Cholestan-3-ol, 2-methylene-, (3Ã¡,5Ã )-" O-Methoxy-Ã -methylbenzyl alcohol "Androstan-17-one, 3-ethyl-3-hydroxy-, (5Ã )-" "1-Ã¡-d-Ribofuranosyl-1,2,4-triazole-3-carboxylic acid" Diisooctyl phthalate Digitoxin Bis(2-ethylhexyl) phthalate ï»¿"Aspidospermidin-17-ol, 1-acetyl-19,21-epoxy-15,16-dimethoxy-" "9,12-Octadecadienoic acid (Z,Z)-" "Fumaric acid, decyl 2-dimethylaminoethyl ester" "3-Octene-2,5-dione, 6,6,7-trimethyl-, (E)-" Pentadecane Ã¡-Sitosterol "Heptadecanoic acid, 15-methyl-, methyl ester" "1,3,2-Dioxaborolane, 4,4-dimethyl-5-oxo-, 2-ethyl" Nicotinic acid hydrazide "Ethanone, 1-(2-hydroxy-5-methylphenyl)-" "Eicosane, 10-methyl-" "Octadecanoic acid, 17-methyl-, methyl ester" "Cholesta-4,6-dien-3-ol, (3Ã¡)-" "Ethanethioic acid, S-[2-(dimethylamino)ethyl] ester" "3-Cyclohexen-1-ol, 3-methyl-" "Phthalic acid, di(2-propylpentyl) ester" "Terephthalic acid, 4-octyl octyl ester" Melezitose "Acethydrazide, 2-(2-benzothiazolylthio)-N2-(3-fluorobenzylideno)-" "1-Phenanthrenecarboxylic acid, tetradecahydro-7-(2-methoxy-2-oxoethylidene)-1,4a,8-trimethyl-9-oxo-, methyl ester, [1S-(1Ã ,4aÃ ,4bÃ¡,8Ã¡,8aÃ ,10aÃ¡)]-" Methyl nicotinate "Undecanoic acid, 10-methyl-, methyl ester" "Docosanoic acid, methyl ester" 5-Octadecenal "Tricyclo[20.8.0.0(7,16)]triacontane, 1(22),7(16)-diepoxy-" "Lupan-3-ol, acetate" "Hexane, 1-(hexyloxy)-2-methyl-" Hexadecane "9-Octadecenoic acid, 1,2,3-propanetriyl ester, (E,E,E)-" Eicosanoic acid 3-Methyl-thiophene-2-carboxamide trans-13-Octadecenoic acid "2-Nonadecanone 2,4-dinitrophenylhydrazine" Methyl 20-methyl-docosanoate "Pseduosarsasapogenin-5,20-dien" 1-Hexadecanol 1-Undecanol "Benzothiazole, 2-(2-hydroxyethylthio)-" "9,19-Cyclolanostan-3-ol, 24-methylene-, (3Ã¡)-" |

**Table S3. Loadings of the variables in the all principal components.**

| **S.No** | **Bolded loadings are highly weighted variables** | **PC1** | **PC2** | **PC3** | **PC4** | **PC5** | **PC6** |
| --- | --- | --- | --- | --- | --- | --- | --- |
|  | **Variation %** | **96.40** | **1.90** |  |  |  |  |
| 1 | (-)-(Z)-Tetrahydro-6-(2-pentenyl)-2H-pyran-2-one | -0.0033 | 0.0165 | -0.0309 | 0.0139 | -0.0040 | 0.9477 |
| 2 | (+)-Aspidospermidine | 0.0006 | 0.0007 | 0.0014 | 0.0004 | 0.0014 | 0.0922 |
| 3 | (2E)-2-dodecen-1-ol | -0.0017 | 0.0044 | -0.0087 | 0.0037 | -0.0005 | -0.1089 |
| 4 | (3beta,4alpha,5alpha,9beta)-4,14-Dimethyl-9,19-cycloergost-24-en-3-ol | **-0.0501** | -0.0100 | 0.0570 | 0.0002 | -0.0385 | -0.1046 |
| 5 | (6E,8E)-4,6,8-Megastigmatriene | -0.0016 | 0.0058 | -0.0116 | 0.0045 | 0.0002 | -0.0828 |
| 6 | (9Z,11S,16S)-1-Acetoxy-9,17-octadecadiene-12,14-diyne-11,16-diol | **0.0706** | -0.0052 | 0.0654 | 0.0352 | 0.0661 | 0.0085 |
| 7 | (E)-3-Octene | 0.0016 | 0.0005 | -0.0026 | -0.0157 | -0.0118 | -0.0026 |
| 8 | (E,E)-11,13-Octadecadien-9-ynoic acid | -0.0020 | 0.0083 | -0.0125 | 0.0094 | -0.0096 | -0.0011 |
| 9 | (E,E)-11,13-Octadecadien-9-ynoic acid.1 | 0.0063 | -0.0213 | -0.0127 | 0.0248 | 0.0008 | 0.0046 |
| 10 | (E,E)-2,4-Decadienal | -0.0008 | 0.0027 | -0.0032 | 0.0038 | -0.0054 | -0.0001 |
| 11 | (Z)-13-Octadecenoic acid | 0.0296 | -0.0251 | 0.3170 | 0.2888 | -0.0214 | 0.0498 |
| 12 | (Z)-2-decenal | 0.0005 | -0.0012 | -0.0002 | 0.0028 | 0.0013 | 0.0005 |
| 13 | 1,25-Dihydroxyvitamin D3-26,23-lactone | -0.0018 | 0.0020 | -0.0063 | 0.0058 | 0.0002 | -0.0001 |
| 14 | 1,3,5,11-Bisabolatetraen-10-one | -0.0111 | 0.0130 | -0.0253 | 0.0128 | -0.0049 | -0.0009 |
| 15 | 1,4-Dimethoxybenzene | -0.0006 | 0.0021 | -0.0037 | 0.0021 | -0.0013 | -0.0006 |
| 16 | 10Z-Nonadecenoic acid | 0.0007 | 0.0042 | 0.0044 | -0.0041 | 0.0017 | -0.0003 |
| 17 | 12,15-Epoxy-13,14-dimethyleicosa-10,12,14-trienoic acid | -0.0084 | 0.0089 | -0.0078 | 0.0166 | -0.0279 | 0.0003 |
| 18 | 12-Methyltridecanoic acid | -0.0027 | 0.0050 | -0.0066 | 0.0070 | -0.0091 | -0.0010 |
| 19 | 13-Heptadecyn-1-ol | -0.0211 | 0.0240 | -0.0409 | 0.0281 | -0.0234 | -0.0070 |
| 20 | 13-Heptadecyn-1-ol.1 | 0.0005 | -0.0019 | -0.0012 | 0.0021 | -0.0001 | 0.0002 |
| 21 | 16b-Hydroxyestradiol | -0.0006 | 0.0010 | -0.0049 | -0.0013 | 0.0069 | 0.0001 |
| 22 | 16-Hydroxyhexadecanoic acid | -0.0248 | 0.0241 | -0.0425 | 0.0283 | -0.0219 | -0.0181 |
| 23 | 17-Octadecynoic acid | -0.0130 | 0.0063 | -0.0335 | 0.0472 | -0.0140 | -0.0028 |
| 24 | 18-methylnonadecanoic acid | 0.0403 | -0.0181 | 0.0180 | 0.0153 | 0.0166 | 0.0074 |
| 25 | 1-Hexadecanethiol | -0.0321 | -0.0131 | 0.0480 | 0.0180 | -0.0727 | 0.0045 |
| 26 | 1-Hexadecanol | 0.0089 | **0.0781** | 0.0792 | -0.1144 | 0.0049 | 0.0000 |
| 27 | 1-hydroperoxy-8-carboxyoctyl-3,4-epoxynon-(2E)-enyl-ether | 0.0005 | 0.0010 | 0.0007 | -0.0031 | -0.0012 | -0.0002 |
| 28 | 1-hydroxylycopene | -0.0109 | **-0.0671** | 0.1507 | -0.0441 | -0.0249 | 0.0074 |
| 29 | 1-Phenylethanol | 0.0031 | 0.0225 | 0.0216 | -0.0269 | 0.0043 | -0.0001 |
| 30 | 1-Undecanol | 0.0026 | 0.0161 | 0.0164 | -0.0170 | 0.0052 | 0.0006 |
| 31 | 2-(2-Amino-1H-benzo[d]imidazol-1-yl)-N-benzyl-8-methoxyquinazolin-4-amine | -0.0053 | 0.0168 | -0.0257 | 0.0193 | -0.0194 | -0.0057 |
| 32 | 2-(Octadecyloxy)ethanol | -0.0080 | -0.0090 | 0.0150 | -0.0028 | -0.0982 | -0.0114 |
| 33 | 2,3,5,8-Tetramethyldecane | -0.0019 | 0.0062 | -0.0158 | 0.0020 | 0.0090 | -0.0009 |
| 34 | 2,4-Di-tert-butylphenol | -0.0055 | 0.0186 | -0.0251 | 0.0238 | -0.0295 | -0.0094 |
| 35 | 2,6,6-Trimethylcyclohex-2-en-1-one | -0.0507 | **-0.0580** | 0.2045 | 0.0458 | -0.2572 | -0.0116 |
| 36 | 2,6-Ditert-butyl-4-[(3,5-ditert-butyl-4-hydroxyphenyl)sulfanylmethylsulfanyl]phenol | -0.0053 | 0.0100 | 0.0111 | 0.0331 | -0.0790 | -0.0038 |
| 37 | 20-methyldocosanoic acid | 0.0058 | 0.0033 | 0.0201 | 0.0381 | 0.0396 | 0.0086 |
| 38 | 24-Methylenecycloartan-3-ol | 0.0383 | -0.0005 | 0.0529 | 0.0699 | 0.0816 | 0.0264 |
| 39 | 2-Decenal | -0.0035 | 0.0178 | -0.0065 | 0.0099 | -0.0187 | -0.0052 |
| 40 | 2'-Hydroxy-4',6'-dimethoxy-3'-methylacetophenone | 0.0010 | 0.0089 | -0.0167 | 0.0440 | -0.0046 | 0.0024 |
| 41 | 2'-Hydroxy-5'-methylacetophenone | 0.0033 | -0.0060 | 0.0038 | 0.0277 | 0.0187 | 0.0052 |
| 42 | 2-Methoxy-4-vinylphenol | 0.0020 | -0.0103 | -0.0145 | 0.0145 | -0.0366 | -0.0012 |
| 43 | 2-Nonadecanone | -0.0008 | 0.0012 | -0.0070 | -0.0025 | 0.0113 | -0.0002 |
| 44 | 2-Nonen-1-ol | -0.0022 | -0.0006 | -0.0226 | -0.0089 | 0.0560 | 0.0033 |
| 45 | 2-Pentadecanone | -0.0084 | 0.0112 | 0.0013 | 0.0289 | -0.0616 | -0.0095 |
| 46 | 3-(4-Methyl-3-pentenyl)thiophene | 0.0028 | 0.0253 | 0.0236 | -0.0302 | 0.0045 | 0.0000 |
| 47 | 3,6-Dimethyl-2(3H)-benzofuranone | -0.0017 | -0.0041 | 0.0110 | 0.0000 | -0.0087 | -0.0001 |
| 48 | 3beta-3-Lupanol | 0.0008 | -0.0020 | 0.0012 | 0.0099 | 0.0067 | 0.0017 |
| 49 | 3beta-Ergosta-5,23-dien-3-ol | -0.0016 | -0.0047 | 0.0114 | -0.0011 | -0.0068 | 0.0006 |
| 50 | 3-Methoxytyramine | -0.0011 | -0.0028 | 0.0083 | 0.0006 | -0.0080 | -0.0004 |
| 51 | 3-Methyl-2,4-nonanedione | -0.0018 | 0.0075 | -0.0099 | 0.0096 | -0.0121 | -0.0014 |
| 52 | 3-Palmitoyl-sn-glycerol | -0.0111 | **0.0414** | -0.1299 | 0.0919 | 0.0158 | -0.0011 |
| 53 | 4-{3-[3-(3,5-dihydroxyphenyl)-6-hydroxy-2-(4-hydroxyphenyl)-2,3-dihydro-1-benzofuran-4-yl]-5-{2-[6-hydroxy-3-(3-hydroxyphenyl)-2-phenyl-2,3-dihydro-1-benzofuran-4-yl]ethenyl}-2,3-dihydro-1-benzofuran-2-yl}benzene-1,2-diol | -0.0013 | 0.0037 | -0.0070 | 0.0031 | -0.0008 | -0.0015 |
| 54 | 4-Ethyl-2-methoxyphenol | 0.0016 | 0.0065 | 0.0074 | -0.0060 | 0.0032 | 0.0013 |
| 55 | 4-Oxononanal | -0.0037 | 0.0007 | -0.0097 | -0.0050 | 0.0192 | -0.0005 |
| 56 | 4-tert-Butylphenol | -0.0040 | 0.0041 | 0.0003 | 0.0108 | -0.0227 | -0.0021 |
| 57 | 5,8,11-Eicosatriynoic acid | -0.0062 | 0.0217 | -0.0257 | 0.0306 | -0.0434 | -0.0054 |
| 58 | 5,8,11-Eicosatriynoic acid.1 | 0.0018 | -0.0053 | -0.0021 | 0.0087 | 0.0026 | 0.0020 |
| 59 | 5alpha-Stigmastan-3,6-dione | -0.0083 | 0.0086 | 0.0041 | 0.0253 | -0.0562 | -0.0037 |
| 60 | 5-Heptyl-2-furanheptanoic acid | -0.0635 | -0.0038 | -0.0340 | -0.0166 | 0.0733 | 0.0017 |
| 61 | 5-Hydroxymethyl-2-furancarboxaldehyde | 0.0070 | -0.0184 | -0.0606 | 0.1047 | 0.0022 | 0.0083 |
| 62 | 6-Octadecenoic acid | **-0.1756** | **0.1398** | -0.2351 | 0.1827 | -0.1712 | -0.0566 |
| 63 | 9,12-Octadecadiynoic acid | -0.0036 | -0.0220 | 0.0434 | -0.0144 | -0.0041 | 0.0041 |
| 64 | 9-Octadecenal | 0.0007 | 0.0006 | -0.0009 | -0.0053 | 0.0047 | -0.0003 |
| 65 | 9Z,12E-Octadecadienoic acid | 0.0611 | -0.0036 | 0.0673 | 0.0639 | 0.0866 | 0.0076 |
| 66 | 9Z,12Z-octadecadienoyl-CoA | -0.0406 | 0.0192 | 0.0110 | 0.0642 | -0.1400 | -0.0324 |
| 67 | Aceteugenol | -0.0028 | -0.0087 | 0.0206 | -0.0025 | -0.0110 | 0.0007 |
| 68 | Acetylhydrazine | **0.0441** | -0.0120 | 0.0398 | 0.0543 | 0.0588 | 0.0127 |
| 69 | Allocholic acid | 0.0076 | 0.0131 | 0.0257 | -0.0139 | 0.0011 | 0.0025 |
| 70 | Allyl glycidyl ether | -0.0220 | 0.0167 | -0.0284 | 0.0220 | -0.0204 | -0.0037 |
| 71 | alpha-Carotene | -0.0002 | -0.0004 | -0.0008 | -0.0014 | 0.0037 | 0.0002 |
| 72 | alpha-Linolenic acid | 0.0220 | 0.0180 | 0.0197 | -0.0027 | -0.0204 | -0.0002 |
| 73 | alpha-Sitosterol | **-0.0510** | 0.0226 | 0.1356 | -0.0220 | 0.2538 | 0.0366 |
| 74 | Androsterone | 0.0039 | 0.0008 | 0.0084 | 0.0141 | 0.0152 | 0.0045 |
| 75 | Arachidic acid | -0.0122 | -0.0029 | 0.0134 | -0.0104 | -0.0448 | -0.0122 |
| 76 | Ascaridole | -0.0009 | -0.0057 | 0.0105 | -0.0043 | 0.0007 | 0.0013 |
| 77 | Ascorbic acid | -0.0257 | **0.2666** | -0.1080 | 0.0949 | -0.0334 | -0.0284 |
| 78 | Behenic acid | 0.0063 | -0.0155 | 0.0021 | 0.0521 | 0.0305 | 0.0098 |
| 79 | Bentonite | -0.0029 | -0.0074 | 0.0197 | -0.0004 | -0.0148 | 0.0017 |
| 80 | Benzene | 0.0184 | 0.0149 | -0.0403 | -0.2015 | -0.1457 | -0.0350 |
| 81 | Benzothiazole | **0.0557** | 0.0223 | 0.0445 | -0.1039 | -0.0301 | 0.0108 |
| 82 | Bis(2-ethylhexyl) phthalate | 0.0169 | -0.0183 | 0.0045 | 0.0401 | 0.0235 | 0.0089 |
| 83 | Bovinic acid | **0.0761** | 0.0227 | 0.0527 | -0.1382 | -0.0466 | 0.0151 |
| 84 | Campesterol | -0.0025 | 0.0301 | -0.0050 | 0.0043 | 0.0345 | 0.0000 |
| 85 | Capric acid | -0.0060 | -0.0066 | 0.0149 | -0.0013 | -0.0085 | 0.0003 |
| 86 | Carnosic acid | 0.0003 | -0.0013 | -0.0022 | -0.0029 | -0.0038 | -0.0007 |
| 87 | Chlordecone | -0.0029 | -0.0030 | 0.0156 | 0.0063 | -0.0258 | -0.0001 |
| 88 | Cholest-5-ene | **-0.0760** | **0.0800** | -0.1492 | 0.0857 | -0.0495 | -0.0313 |
| 89 | Cholesta-4,6-dien-3-one | -0.0018 | 0.0011 | 0.0053 | 0.0072 | -0.0194 | 0.0008 |
| 90 | Cholestan-3-ol | **-0.0876** | 0.0093 | -0.1255 | -0.0490 | 0.2048 | -0.0053 |
| 91 | Cholesterol | -0.0093 | 0.0070 | 0.0126 | 0.0287 | -0.0702 | -0.0075 |
| 92 | cis-4-Decenol | -0.0099 | -0.0247 | 0.0457 | -0.0167 | 0.0000 | 0.0074 |
| 93 | cis-Vaccenic acid | **-0.6285** | **-0.2402** | 0.2406 | -0.0873 | 0.2433 | -0.0992 |
| 94 | Clionasterol | **0.0452** | **-0.0835** | 0.0120 | 0.2473 | 0.1430 | 0.0495 |
| 95 | D-Fructose | 0.0007 | 0.0020 | 0.0013 | -0.0061 | -0.0025 | -0.0009 |
| 96 | Digitoxin | 0.0007 | 0.0015 | 0.0021 | -0.0007 | 0.0015 | 0.0004 |
| 97 | Dihydro-6-isopropyl-2,4-dimethyl-4H-1,3,5-dithiazine | -0.0007 | -0.0006 | 0.0021 | 0.0004 | -0.0026 | 0.0000 |
| 98 | Dihydrobrassicasterol | **0.0654** | **-0.1122** | -0.0851 | 0.1085 | -0.0973 | -0.0163 |
| 99 | Dodecanoic acid | -0.0318 | -0.0100 | 0.0103 | -0.1142 | 0.2077 | 0.0123 |
| 100 | Eicosane | -0.0342 | 0.0130 | -0.0508 | 0.0092 | 0.0454 | -0.0147 |
| 101 | Elaidic acid | -0.0289 | 0.0307 | 0.1413 | -0.0632 | 0.0408 | 0.0160 |
| 102 | Ergost-4-en-3-one | -0.0080 | -0.0038 | 0.0209 | 0.0104 | -0.0379 | 0.0016 |
| 103 | Ethanethioic acid | 0.0118 | -0.0149 | 0.0024 | 0.0340 | 0.0191 | 0.0097 |
| 104 | Fumaric acid | 0.0096 | -0.0138 | 0.0110 | 0.0653 | 0.0457 | 0.0104 |
| 105 | gamma-Ionone | -0.0004 | -0.0008 | -0.0005 | -0.0022 | 0.0052 | 0.0004 |
| 106 | gamma-Tocopherol | 0.0051 | 0.0080 | 0.0024 | -0.0279 | -0.0099 | -0.0038 |
| 107 | Geranylgeranylcysteine | -0.0040 | -0.0012 | 0.0012 | -0.0006 | 0.0009 | -0.0003 |
| 108 | Geranylgeranyl-PP | 0.0074 | **-0.0634** | -0.0330 | 0.0293 | -0.0507 | -0.0064 |
| 109 | Heneicosane | -0.0711 | -0.1103 | 0.1379 | -0.1193 | 0.1539 | 0.0513 |
| 110 | Heneicosanoic acid | 0.0045 | -0.0275 | 0.0446 | -0.0071 | -0.1428 | -0.0074 |
| 111 | Heptadecane | -0.0021 | 0.0034 | 0.0270 | -0.0124 | 0.0345 | 0.0033 |
| 112 | Heptadecanoic acid | 0.0370 | **-0.0726** | 0.0395 | -0.1928 | -0.1579 | -0.0286 |
| 113 | Hexacosanoic acid | -0.0089 | -0.0089 | 0.0404 | -0.0336 | 0.0538 | 0.0069 |
| 114 | Hexadecane | 0.0038 | -0.0068 | -0.0038 | -0.0090 | -0.0094 | -0.0009 |
| 115 | Hexane | 0.0003 | -0.0008 | -0.0009 | -0.0008 | -0.0014 | -0.0003 |
| 116 | Isofucosterol 3-O-[6-O-(9,12-Octadecadienoyl)-b-D-glucopyranoside] | **0.0436** | **0.1756** | 0.1254 | 0.1212 | 0.0473 | 0.0349 |
| 117 | isosorbide | 0.0010 | 0.0006 | -0.0023 | 0.0052 | -0.0021 | -0.0002 |
| 118 | L-galacto-2-Heptulose | -0.0039 | -0.0047 | 0.0086 | -0.0027 | -0.0007 | 0.0022 |
| 119 | Lignocerane | **-0.0572** | **-0.0400** | 0.0292 | -0.0503 | 0.0928 | 0.0121 |
| 120 | Linoleic acid | **-0.0736** | -0.0236 | -0.1165 | -0.0626 | 0.2288 | 0.0242 |
| 121 | Lycophyll | 0.0005 | -0.0012 | -0.0002 | 0.0028 | 0.0013 | 0.0005 |
| 122 | Melezitose | 0.0008 | -0.0016 | -0.0017 | -0.0016 | -0.0027 | -0.0004 |
| 123 | Methyl hexadecanoic acid | **-0.2937** | -0.1011 | 0.2711 | 0.0736 | -0.3327 | 0.0469 |
| 124 | Methyl nicotinate | 0.0009 | 0.0004 | 0.0048 | 0.0122 | 0.0114 | 0.0016 |
| 125 | Methyl stearate | 0.0133 | -0.0955 | 0.0549 | 0.1944 | -0.0137 | 0.0319 |
| 126 | Methyl tetradecanoate | 0.0009 | -0.0123 | 0.0292 | 0.0117 | 0.0220 | 0.0071 |
| 127 | Myristic acid | -0.0301 | 0.0223 | -0.0457 | -0.1007 | 0.3031 | 0.0283 |
| 128 | Myristoleic acid | -0.0003 | 0.0008 | 0.0080 | -0.0049 | 0.0025 | 0.0005 |
| 129 | Nicotinic acid | 0.0014 | -0.0036 | -0.0006 | 0.0084 | 0.0039 | 0.0011 |
| 130 | Nonadecane | -0.0279 | 0.0002 | 0.0092 | 0.0161 | -0.0376 | -0.0072 |
| 131 | Nonanal | -0.0015 | 0.0042 | -0.0065 | 0.0049 | -0.0049 | -0.0012 |
| 132 | N-Triacontane | 0.0048 | 0.0113 | 0.0181 | 0.0020 | 0.0172 | 0.0015 |
| 133 | Octadec-9-enoic Acid | -0.0256 | -0.0140 | 0.0376 | 0.0059 | -0.0393 | 0.0010 |
| 134 | Octadecane | 0.0042 | -0.0136 | -0.0124 | 0.0005 | -0.0124 | -0.0017 |
| 135 | Oleamide | -0.0269 | -0.0213 | 0.0288 | -0.0174 | 0.0179 | -0.0049 |
| 136 | Oleic acid | -0.0042 | **-0.7478** | -0.1592 | -0.0829 | -0.1223 | 0.0626 |
| 137 | Palmitelaidic acid | -0.0009 | -0.0025 | 0.0044 | -0.0020 | 0.0009 | 0.0008 |
| 138 | Palmitic acid | **0.2779** | **0.1075** | 0.3122 | -0.4906 | -0.0537 | 0.0265 |
| 139 | Palmitoleic acid | 0.0043 | -0.0040 | 0.0007 | 0.0056 | 0.0029 | 0.0010 |
| 140 | Palmitoyl chloride | 0.0119 | **-0.0402** | -0.0113 | 0.0188 | -0.0658 | -0.0014 |
| 141 | Panaxydol | 0.0005 | -0.0017 | -0.0015 | 0.0003 | -0.0014 | 0.0000 |
| 142 | Paullinic acid | **0.0607** | **0.0548** | 0.0134 | -0.0814 | -0.0880 | -0.0182 |
| 143 | Pentadecane | 0.0011 | -0.0024 | -0.0003 | 0.0055 | 0.0027 | 0.0008 |
| 144 | Pentadecanoic acid | **-0.0520** | 0.1463 | 0.3692 | 0.0458 | -0.1016 | 0.0116 |
| 145 | Pentatriacontane | -0.0272 | -0.0014 | -0.0417 | -0.0281 | 0.0985 | 0.0053 |
| 146 | petroselinate | **0.2989** | **-0.2517** | -0.0578 | -0.0630 | -0.1592 | -0.0173 |
| 147 | Phenylacetaldehyde | -0.0004 | 0.0014 | -0.0021 | 0.0016 | -0.0016 | -0.0002 |
| 148 | Phthalic acid | 0.0108 | **0.0350** | -0.0078 | 0.0692 | -0.0174 | -0.0010 |
| 149 | Pinolidoxin | -0.0004 | -0.0025 | 0.0045 | -0.0019 | 0.0005 | 0.0005 |
| 150 | Ribavirin | 0.0040 | -0.0071 | -0.0053 | -0.0013 | -0.0067 | -0.0002 |
| 151 | Sarsasapogenin | 0.0006 | 0.0022 | 0.0011 | -0.0068 | -0.0030 | -0.0009 |
| 152 | Solketal | -0.0006 | -0.0019 | 0.0031 | -0.0017 | 0.0012 | 0.0003 |
| 153 | Squalene | 0.0053 | 0.0141 | 0.0908 | -0.0057 | 0.0264 | 0.0108 |
| 154 | Stearaldehyde | -0.0008 | -0.0013 | 0.0035 | 0.0001 | -0.0029 | 0.0002 |
| 155 | Stearic acid | 0.0123 | 0.0139 | 0.0155 | -0.0403 | -0.0112 | -0.0035 |
| 156 | Stigmasterol | **0.0407** | -0.0180 | -0.2356 | -0.2931 | 0.0803 | -0.0375 |
| 157 | Terephthalic acid | 0.0345 | 0.0316 | 0.0250 | -0.1460 | -0.0680 | -0.0304 |
| 158 | Tetracosanoic acid | 0.0032 | 0.0043 | -0.0263 | 0.0016 | -0.0393 | -0.0052 |
| 159 | Tetradec-2-enal | -0.0006 | 0.0016 | -0.0043 | 0.0004 | 0.0028 | -0.0003 |
| 160 | Tetradecane | 0.0031 | -0.0091 | -0.0044 | 0.0128 | 0.0025 | 0.0014 |
| 161 | trans-12-Octadecenoic acid | **0.4726** | **-0.2162** | 0.2412 | 0.3028 | 0.2947 | -0.1344 |
| 162 | Tridecanedioyl-CoA | -0.0005 | -0.0032 | 0.0059 | -0.0023 | 0.0002 | 0.0006 |
| 163 | Tridecanoic acid | -0.0252 | -0.0187 | 0.1207 | -0.0482 | 0.0521 | 0.0170 |
| 164 | Undecanoic acid | -0.0051 | 0.0106 | -0.0289 | 0.0025 | 0.0190 | -0.0050 |
| 165 | Undecanoic acid.1 | 0.0053 | -0.0151 | -0.0142 | -0.0030 | -0.0164 | -0.0017 |
| 166 | Vitamin E Nicotinate | 0.0054 | 0.0091 | 0.0063 | -0.0319 | -0.0136 | -0.0042 |
| 167 | xi-3-Methyl-3-cyclohexen-1-ol | 0.0029 | 0.0118 | 0.0168 | 0.0008 | 0.0157 | 0.0023 |

**Table S4. Loadings of the variables in Orthogonal Partial Least square discrimination**

| **S.No** | **Compound name** | **Loading (t1)** | **OrthoLoading (to1)** |
| --- | --- | --- | --- |
| 1 | (-)-(Z)-Tetrahydro-6-(2-pentenyl)-2H-pyran-2-one | -0.0655 | 0.0868 |
| 2 | (+)-Aspidospermidine | 0.0890 | -0.0339 |
| 3 | (2E)-2-dodecen-1-ol | -0.0821 | 0.0593 |
| 4 | (3beta,4alpha,5alpha,9beta)-4,14-Dimethyl-9,19-cycloergost-24-en-3-ol | -0.0917 | -0.0302 |
| 5 | (6E,8E)-4,6,8-Megastigmatriene | -0.0743 | 0.0772 |
| 6 | (9Z,11S,16S)-1-Acetoxy-9,17-octadecadiene-12,14-diyne-11,16-diol | 0.0927 | 0.0023 |
| 7 | (E)-3-Octene | 0.0698 | -0.0639 |
| 8 | (E,E)-11,13-Octadecadien-9-ynoic acid | -0.0723 | 0.0593 |
| 9 | (E,E)-11,13-Octadecadien-9-ynoic acid.1 | 0.0795 | 0.1128 |
| 10 | (E,E)-2,4-Decadienal | -0.0755 | 0.0358 |
| 11 | (Z)-13-Octadecenoic acid | 0.0594 | -0.0465 |
| 12 | (Z)-2-decenal | 0.0783 | 0.1036 |
| 13 | 1,25-Dihydroxyvitamin D3-26,23-lactone | -0.0876 | 0.0650 |
| 14 | 1,3,5,11-Bisabolatetraen-10-one | -0.0911 | 0.0251 |
| 15 | 1,4-Dimethoxybenzene | -0.0760 | 0.0648 |
| 16 | 10Z-Nonadecenoic acid | 0.0628 | -0.1555 |
| 17 | 12,15-Epoxy-13,14-dimethyleicosa-10,12,14-trienoic acid | -0.0902 | 0.0024 |
| 18 | 12-Methyltridecanoic acid | -0.0873 | 0.0231 |
| 19 | 13-Heptadecyn-1-ol | -0.0913 | 0.0196 |
| 20 | 13-Heptadecyn-1-ol.1 | 0.0789 | 0.1147 |
| 21 | 16b-Hydroxyestradiol | -0.0664 | 0.0869 |
| 22 | 16-Hydroxyhexadecanoic acid | -0.0919 | 0.0166 |
| 23 | 17-Octadecynoic acid | -0.0887 | 0.0593 |
| 24 | 18-methylnonadecanoic acid | 0.0928 | 0.0130 |
| 25 | 1-Hexadecanethiol | -0.0904 | -0.0324 |
| 26 | 1-Hexadecanol | 0.0468 | -0.1922 |
| 27 | 1-hydroperoxy-8-carboxyoctyl-3,4-epoxynon-(2E)-enyl-ether | 0.0783 | -0.1036 |
| 28 | 1-hydroxylycopene | -0.0451 | -0.1224 |
| 29 | 1-Phenylethanol | 0.0558 | -0.1722 |
| 30 | 1-Undecanol | 0.0619 | -0.1587 |
| 31 | 2-(2-Amino-1H-benzo[d]imidazol-1-yl)-N-benzyl-8-methoxyquinazolin-4-amine | -0.0787 | 0.0503 |
| 32 | 2-(Octadecyloxy)ethanol | -0.0735 | -0.0563 |
| 33 | 2,3,5,8-Tetramethyldecane | -0.0718 | 0.0895 |
| 34 | 2,4-Di-tert-butylphenol | -0.0769 | 0.0436 |
| 35 | 2,6,6-Trimethylcyclohex-2-en-1-one | -0.0813 | -0.0665 |
| 36 | 2,6-Ditert-butyl-4-[(3,5-ditert-butyl-4-hydroxyphenyl)sulfanylmethylsulfanyl]phenol | -0.0653 | -0.0378 |
| 37 | 20-methyldocosanoic acid | 0.0749 | 0.0229 |
| 38 | 24-Methylenecycloartan-3-ol | 0.0908 | 0.0103 |
| 39 | 2-Decenal | -0.0749 | -0.0234 |
| 40 | 2'-Hydroxy-4',6'-dimethoxy-3'-methylacetophenone | 0.0164 | 0.1514 |
| 41 | 2'-Hydroxy-5'-methylacetophenone | 0.0726 | 0.0904 |
| 42 | 2-Methoxy-4-vinylphenol | 0.0481 | 0.1204 |
| 43 | 2-Nonadecanone | -0.0597 | 0.0905 |
| 44 | 2-Nonen-1-ol | -0.0461 | 0.1047 |
| 45 | 2-Pentadecanone | -0.0830 | -0.0157 |
| 46 | 3-(4-Methyl-3-pentenyl)thiophene | 0.0476 | -0.1852 |
| 47 | 3,6-Dimethyl-2(3H)-benzofuranone | -0.0717 | -0.0896 |
| 48 | 3beta-3-Lupanol | 0.0601 | 0.1092 |
| 49 | 3beta-Ergosta-5,23-dien-3-ol | -0.0691 | -0.0934 |
| 50 | 3-Methoxytyramine | -0.0664 | -0.0977 |
| 51 | 3-Methyl-2,4-nonanedione | -0.0708 | 0.0496 |
| 52 | 3-Palmitoyl-sn-glycerol | -0.0598 | 0.1425 |
| 53 | 4-{3-[3-(3,5-dihydroxyphenyl)-6-hydroxy-2-(4-hydroxyphenyl)-2,3-dihydro-1-benzofuran-4-yl]-5-{2-[6-hydroxy-3-(3-hydroxyphenyl)-2-phenyl-2,3-dihydro-1-benzofuran-4-yl]ethenyl}-2,3-dihydro-1-benzofuran-2-yl}benzene-1,2-diol | -0.0812 | 0.0603 |
| 54 | 4-Ethyl-2-methoxyphenol | 0.0749 | -0.1233 |
| 55 | 4-Oxononanal | -0.0864 | 0.0348 |
| 56 | 4-tert-Butylphenol | -0.0866 | -0.0140 |
| 57 | 5,8,11-Eicosatriynoic acid | -0.0746 | 0.0365 |
| 58 | 5,8,11-Eicosatriynoic acid.1 | 0.0806 | 0.1053 |
| 59 | 5alpha-Stigmastan-3,6-dione | -0.0844 | -0.0203 |
| 60 | 5-Heptyl-2-furanheptanoic acid | -0.0925 | 0.0013 |
| 61 | 5-Hydroxymethyl-2-furancarboxaldehyde | 0.0483 | 0.1985 |
| 62 | 6-Octadecenoic acid | -0.0923 | 0.0111 |
| 63 | 9,12-Octadecadiynoic acid | -0.0479 | -0.1104 |
| 64 | 9-Octadecenal | 0.0737 | -0.0292 |
| 65 | 9Z,12E-Octadecadienoic acid | 0.0921 | 0.0060 |
| 66 | 9Z,12Z-octadecadienoyl-CoA | -0.0907 | -0.0153 |
| 67 | Aceteugenol | -0.0673 | -0.0958 |
| 68 | Acetylhydrazine | 0.0920 | 0.0139 |
| 69 | Allocholic acid | 0.0867 | -0.0844 |
| 70 | Allyl glycidyl ether | -0.0924 | 0.0104 |
| 71 | alpha-Carotene | -0.0589 | 0.0407 |
| 72 | alpha-Linolenic acid | 0.0918 | -0.0207 |
| 73 | alpha-Sitosterol | -0.0852 | -0.0491 |
| 74 | Androsterone | 0.0859 | 0.0163 |
| 75 | Arachidic acid | -0.0895 | -0.0456 |
| 76 | Ascaridole | -0.0499 | -0.1038 |
| 77 | Ascorbic acid | -0.0549 | -0.0197 |
| 78 | Behenic acid | 0.0722 | 0.1060 |
| 79 | Bentonite | -0.0705 | -0.0916 |
| 80 | Benzene | 0.0655 | -0.0680 |
| 81 | Benzothiazole | 0.0919 | -0.0298 |
| 82 | Bis(2-ethylhexyl) phthalate | 0.0900 | 0.0465 |
| 83 | Bovinic acid | 0.0921 | -0.0264 |
| 84 | Campesterol | -0.0461 | -0.0331 |
| 85 | Capric acid | -0.0879 | -0.0452 |
| 86 | Carnosic acid | 0.0544 | 0.0352 |
| 87 | Chlordecone | -0.0725 | -0.0791 |
| 88 | Cholest-5-ene | -0.0916 | 0.0204 |
| 89 | Cholesta-4,6-dien-3-one | -0.0735 | -0.0514 |
| 90 | Cholestan-3-ol | -0.0914 | 0.0156 |
| 91 | Cholesterol | -0.0826 | -0.0322 |
| 92 | cis-4-Decenol | -0.0778 | -0.0683 |
| 93 | cis-Vaccenic acid | -0.0922 | -0.0122 |
| 94 | Clionasterol | 0.0815 | 0.0836 |
| 95 | D-Fructose | 0.0717 | -0.1221 |
| 96 | Digitoxin | 0.0850 | -0.0743 |
| 97 | Dihydro-6-isopropyl-2,4-dimethyl-4H-1,3,5-dithiazine | -0.0851 | -0.0559 |
| 98 | Dihydrobrassicasterol | 0.0884 | 0.0652 |
| 99 | Dodecanoic acid | -0.0821 | -0.0253 |
| 100 | Eicosane | -0.0923 | 0.0184 |
| 101 | Elaidic acid | -0.0807 | -0.1159 |
| 102 | Ergost-4-en-3-one | -0.0858 | -0.0491 |
| 103 | Ethanethioic acid | 0.0888 | 0.0549 |
| 104 | Fumaric acid | 0.0777 | 0.0772 |
| 105 | gamma-Ionone | -0.0625 | 0.0195 |
| 106 | gamma-Tocopherol | 0.0833 | -0.0727 |
| 107 | Geranylgeranylcysteine | -0.0924 | -0.0124 |
| 108 | Geranylgeranyl-PP | 0.0551 | 0.1315 |
| 109 | Heneicosane | -0.0861 | -0.0334 |
| 110 | Heneicosanoic acid | 0.0394 | -0.0825 |
| 111 | Heptadecane | -0.0449 | -0.1433 |
| 112 | Heptadecanoic acid | 0.0826 | -0.0435 |
| 113 | Hexacosanoic acid | -0.0749 | -0.0832 |
| 114 | Hexadecane | 0.0880 | 0.0183 |
| 115 | Hexane | 0.0802 | 0.0510 |
| 116 | Isofucosterol 3-O-[6-O-(9,12-Octadecadienoyl)-b-D-glucopyranoside] | 0.0767 | -0.0522 |
| 117 | isosorbide | 0.0803 | 0.0822 |
| 118 | L-galacto-2-Heptulose | -0.0881 | -0.0405 |
| 119 | Lignocerane | -0.0908 | -0.0140 |
| 120 | Linoleic acid | -0.0902 | 0.0230 |
| 121 | Lycophyll | 0.0783 | 0.1036 |
| 122 | Melezitose | 0.0853 | 0.0401 |
| 123 | Methyl hexadecanoic acid | -0.0918 | -0.0231 |
| 124 | Methyl nicotinate | 0.0544 | 0.0491 |
| 125 | Methyl stearate | 0.0503 | 0.0935 |
| 126 | Methyl tetradecanoate | 0.0282 | -0.0462 |
| 127 | Myristic acid | -0.0764 | 0.0041 |
| 128 | Myristoleic acid | -0.0259 | -0.2216 |
| 129 | Nicotinic acid | 0.0783 | 0.1036 |
| 130 | Nonadecane | -0.0925 | -0.0144 |
| 131 | Nonanal | -0.0816 | 0.0454 |
| 132 | N-Triacontane | 0.0830 | -0.0651 |
| 133 | Octadec-9-enoic Acid | -0.0907 | -0.0315 |
| 134 | Octadecane | 0.0811 | 0.0854 |
| 135 | Oleamide | -0.0907 | -0.0233 |
| 136 | Oleic acid | -0.0013 | 0.1079 |
| 137 | Palmitelaidic acid | -0.0746 | -0.0704 |
| 138 | Palmitic acid | 0.0920 | -0.0333 |
| 139 | Palmitoleic acid | 0.0917 | 0.0339 |
| 140 | Palmitoyl chloride | 0.0801 | 0.0603 |
| 141 | Panaxydol | 0.0783 | 0.0988 |
| 142 | Paullinic acid | 0.0912 | -0.0234 |
| 143 | Pentadecane | 0.0824 | 0.0892 |
| 144 | Pentadecanoic acid | -0.0723 | -0.1363 |
| 145 | Pentatriacontane | -0.0898 | 0.0183 |
| 146 | petroselinate | 0.0924 | 0.0206 |
| 147 | Phenylacetaldehyde | -0.0787 | 0.0503 |
| 148 | Phthalic acid | 0.0736 | 0.0340 |
| 149 | Pinolidoxin | -0.0510 | -0.1015 |
| 150 | Ribavirin | 0.0892 | 0.0470 |
| 151 | Sarsasapogenin | 0.0619 | -0.1391 |
| 152 | Solketal | -0.0713 | -0.0710 |
| 153 | Squalene | 0.0519 | -0.1711 |
| 154 | Stearaldehyde | -0.0797 | -0.0724 |
| 155 | Stearic acid | 0.0889 | -0.0603 |
| 156 | Stigmasterol | 0.0722 | 0.0415 |
| 157 | Terephthalic acid | 0.0873 | -0.0599 |
| 158 | Tetracosanoic acid | 0.0601 | 0.0734 |
| 159 | Tetradec-2-enal | -0.0757 | 0.0817 |
| 160 | Tetradecane | 0.0815 | 0.1037 |
| 161 | trans-12-Octadecenoic acid | 0.0927 | 0.0153 |
| 162 | Tridecanedioyl-CoA | -0.0491 | -0.1056 |
| 163 | Tridecanoic acid | -0.0806 | -0.0898 |
| 164 | Trigonelline | 0.0474 | -0.1858 |
| 165 | Undecanoic acid | -0.0809 | 0.0695 |
| 166 | Undecanoic acid.1 | 0.0829 | 0.0716 |
| 167 | Vitamin E Nicotinate | 0.0819 | -0.0879 |
| 168 | xi-3-Methyl-3-cyclohexen-1-ol | 0.0716 | -0.0985 |

**Table S5. Volcano plot analysis of differential metabolites**

| **S.No** | **Compound name** | **FC** | **log2(FC)** | **raw.pval** | **=-LOG10(p)** |
| --- | --- | --- | --- | --- | --- |
| 1 | 18-methylnonadecanoic acid | 0.19049 | -2.3922 | 8.46E-06 | 5.0727 |
| 2 | trans-12-Octadecenoic acid | 0.18891 | -2.4042 | 2.50E-05 | 4.6026 |
| 3 | (9Z,11S,16S)-1-Acetoxy-9,17-octadecadiene-12,14-diyne-11,16-diol | 0.18776 | -2.4131 | 2.59E-05 | 4.587 |
| 4 | 5-Heptyl-2-furanheptanoic acid | 5.3991 | 2.4327 | 2.99E-05 | 4.5242 |
| 5 | Geranylgeranylcysteine | 5.3704 | 2.425 | 5.80E-05 | 4.2365 |
| 6 | Nonadecane | 5.4918 | 2.4573 | 6.19E-05 | 4.2083 |
| 7 | petroselinate | 0.18392 | -2.4428 | 7.07E-05 | 4.1505 |
| 8 | Eicosane | 30.071 | 4.9103 | 8.36E-05 | 4.0779 |
| 9 | Allyl glycidyl ether | 5.4592 | 2.4487 | 9.22E-05 | 4.0355 |
| 10 | cis-Vaccenic acid | 62.008 | 5.9544 | 9.26E-05 | 4.0333 |
| 11 | 6-Octadecenoic acid | 5.4912 | 2.4571 | 0.0001164 | 3.934 |
| 12 | Bovinic acid | 0.17869 | -2.4845 | 0.0001575 | 3.8027 |
| 13 | 9Z,12E-Octadecadienoic acid | 0.18143 | -2.4625 | 0.00016218 | 3.79 |
| 14 | Acetylhydrazine | 0.18562 | -2.4295 | 0.00017663 | 3.7529 |
| 15 | Palmitic acid | 0.13412 | -2.8984 | 0.00018889 | 3.7238 |
| 16 | Benzothiazole | 0.17836 | -2.4872 | 0.00020679 | 3.6845 |
| 17 | Methyl hexadecanoic acid | 5.5732 | 2.4785 | 0.00027522 | 3.5603 |
| 18 | 16-Hydroxyhexadecanoic acid | 5.7143 | 2.5146 | 0.00029474 | 3.5306 |
| 19 | Palmitoleic acid | 0.17419 | -2.5212 | 0.00030052 | 3.5221 |
| 20 | (3beta,4alpha,5alpha,9beta)-4,14-Dimethyl-9,19-cycloergost-24-en-3-ol | 5.9746 | 2.5788 | 0.0003068 | 3.5131 |
| 21 | Cholestan-3-ol | 130.75 | 7.0307 | 0.00037742 | 3.4232 |
| 22 | Cholest-5-ene | 5.86 | 2.5509 | 0.0004401 | 3.3564 |
| 23 | Paullinic acid | 0.25803 | -1.9544 | 0.00054461 | 3.2639 |
| 24 | 13-Heptadecyn-1-ol | 5.8915 | 2.5586 | 0.00063034 | 3.2004 |
| 25 | Lignocerane | 6.021 | 2.59 | 0.0007121 | 3.1475 |
| 26 | 1,3,5,11-Bisabolatetraen-10-one | 6.0606 | 2.5995 | 0.00075517 | 3.122 |
| 27 | Oleamide | 5.8488 | 2.5481 | 0.00085212 | 3.0695 |
| 28 | 24-Methylenecycloartan-3-ol | 0.17143 | -2.5443 | 0.00089408 | 3.0486 |
| 29 | Octadec-9-enoic Acid | 5.8414 | 2.5463 | 0.0009615 | 3.017 |
| 30 | 9Z,12Z-octadecadienoyl-CoA | 6.1432 | 2.619 | 0.001083 | 2.9654 |
| 31 | Linoleic acid | 7.0417 | 2.8159 | 0.0011988 | 2.9212 |
| 32 | 1-Hexadecanethiol | 6.0976 | 2.6082 | 0.0012097 | 2.9173 |
| 33 | Bis(2-ethylhexyl) phthalate | 0.16387 | -2.6094 | 0.0015791 | 2.8016 |
| 34 | 12,15-Epoxy-13,14-dimethyleicosa-10,12,14-trienoic acid | 5.7619 | 2.5265 | 0.0015885 | 2.799 |
| 35 | Pentatriacontane | 5.9722 | 2.5783 | 0.0016281 | 2.7883 |
| 36 | Arachidic acid | 6.9583 | 2.7987 | 0.0021693 | 2.6637 |
| 37 | Ribavirin | 0.16071 | -2.6374 | 0.0025616 | 2.5915 |
| 38 | (+)-Aspidospermidine | 0.14815 | -2.7549 | 0.0028102 | 2.5513 |
| 39 | Stearic acid | 0.15862 | -2.6563 | 0.0029827 | 2.5254 |
| 40 | Ethanethioic acid | 0.15636 | -2.677 | 0.0030363 | 2.5177 |
| 41 | 17-Octadecynoic acid | 2.0129 | 1.0093 | 0.003489 | 2.4573 |
| 42 | Dihydrobrassicasterol | 0.047336 | -4.4009 | 0.0036658 | 2.4358 |
| 43 | L-galacto-2-Heptulose | 6 | 2.585 | 0.0039779 | 2.4003 |
| 44 | Hexadecane | 0.066667 | -3.9069 | 0.0043649 | 2.36 |
| 45 | Capric acid | 6.087 | 2.6057 | 0.0044224 | 2.3543 |
| 46 | Terephthalic acid | 0.14088 | -2.8275 | 0.0055717 | 2.254 |
| 47 | 12-Methyltridecanoic acid | 6.5 | 2.7004 | 0.0061525 | 2.2109 |
| 48 | Allocholic acid | 0.10784 | -3.213 | 0.0066591 | 2.1766 |
| 49 | 4-Oxononanal | 6.1905 | 2.6301 | 0.0069681 | 2.1569 |
| 50 | Heneicosane | 7.1772 | 2.8434 | 0.0075002 | 2.1249 |
| 51 | 4-tert-Butylphenol | 7.0513 | 2.8179 | 0.0077728 | 2.1094 |
| 52 | Androsterone | 0.14444 | -2.7914 | 0.008707 | 2.0601 |
| 53 | Ergost-4-en-3-one | 7.7536 | 2.9549 | 0.0090939 | 2.0412 |
| 54 | Melezitose | 0.16364 | -2.6114 | 0.010031 | 1.9987 |
| 55 | Digitoxin | 0.13333 | -2.9069 | 0.010795 | 1.9668 |
| 56 | Dihydro-6-isopropyl-2,4-dimethyl-4H-1,3,5-dithiazine | 7.5 | 2.9069 | 0.010795 | 1.9668 |
| 57 | 5alpha-Stigmastan-3,6-dione | 7.8472 | 2.9722 | 0.013523 | 1.8689 |
| 58 | gamma-Tocopherol | 0.13043 | -2.9386 | 0.015774 | 1.8021 |
| 59 | N-Triacontane | 0.1125 | -3.152 | 0.016983 | 1.77 |
| 60 | Undecanoic acid.1 | 0.13333 | -2.9069 | 0.017091 | 1.7672 |
| 61 | 2-Pentadecanone | 7.9861 | 2.9975 | 0.018137 | 1.7414 |
| 62 | Dodecanoic acid | 4.1092 | 2.0389 | 0.018564 | 1.7313 |
| 63 | Pentadecane | 0.12 | -3.0589 | 0.018932 | 1.7228 |
| 64 | Cholesterol | 8.9855 | 3.1676 | 0.019327 | 1.7138 |
| 65 | Vitamin E Nicotinate | 0.11507 | -3.1194 | 0.020412 | 1.6901 |
| 66 | (2E)-2-dodecen-1-ol | 9.5833 | 3.2605 | 0.020838 | 1.6811 |
| 67 | Clionasterol | 0.12533 | -2.9962 | 0.02217 | 1.6542 |
| 68 | Tetradecane | 0.10244 | -3.2872 | 0.022238 | 1.6529 |
| 69 | Nonanal | 8.75 | 3.1293 | 0.023177 | 1.635 |
| 70 | 2,6,6-Trimethylcyclohex-2-en-1-one | 8.9431 | 3.1608 | 0.023216 | 1.6342 |
| 71 | Octadecane | 0.11786 | -3.0849 | 0.023673 | 1.6257 |
| 72 | 4-{3-[3-(3,5-dihydroxyphenyl)-6-hydroxy-2-(4-hydroxyphenyl)-2,3-dihydro-1-benzofuran-4-yl]-5-{2-[6-hydroxy-3-(3-hydroxyphenyl)-2-phenyl-2,3-dihydro-1-benzofuran-4-yl]ethenyl}-2,3-dihydro-1-benzofuran-2-yl}benzene-1,2-diol | 10 | 3.3219 | 0.024119 | 1.6176 |
| 73 | Tridecanoic acid | 3.2154 | 1.685 | 0.024542 | 1.6101 |
| 74 | Undecanoic acid | 9.5833 | 3.2605 | 0.024546 | 1.61 |
| 75 | 5,8,11-Eicosatriynoic acid.1 | 0.1 | -3.3219 | 0.025847 | 1.5876 |
| 76 | Palmitoyl chloride | 0.48352 | -1.0484 | 0.027183 | 1.5657 |
| 77 | Hexane | 0.15 | -2.737 | 0.027277 | 1.5642 |
| 78 | Stearaldehyde | 8.3333 | 3.0589 | 0.029212 | 1.5344 |
| 79 | (E,E)-11,13-Octadecadien-9-ynoic acid.1 | 0.088889 | -3.4919 | 0.03057 | 1.5147 |
| 80 | 13-Heptadecyn-1-ol.1 | 0.085714 | -3.5443 | 0.032901 | 1.4828 |
| 81 | (Z)-2-decenal | 0.1 | -3.3219 | 0.035606 | 1.4485 |
| 82 | 1-hydroperoxy-8-carboxyoctyl-3,4-epoxynon-(2E)-enyl-ether | 0.1 | -3.3219 | 0.035606 | 1.4485 |
| 83 | 2-(2-Amino-1H-benzo[d]imidazol-1-yl)-N-benzyl-8-methoxyquinazolin-4-amine | 10 | 3.3219 | 0.035606 | 1.4485 |
| 84 | Lycophyll | 0.1 | -3.3219 | 0.035606 | 1.4485 |
| 85 | Nicotinic acid | 0.1 | -3.3219 | 0.035606 | 1.4485 |
| 86 | Panaxydol | 0.1 | -3.3219 | 0.035606 | 1.4485 |
| 87 | Phenylacetaldehyde | 10 | 3.3219 | 0.035606 | 1.4485 |
| 88 | cis-4-Decenol | 7.6786 | 2.9408 | 0.036763 | 1.4346 |
| 89 | Fumaric acid | 0.12923 | -2.952 | 0.038791 | 1.4113 |
| 90 | Isofucosterol 3-O-[6-O-(9,12-Octadecadienoyl)-b-D-glucopyranoside] | 0.49153 | -1.0247 | 0.042495 | 1.3717 |
| 91 | Myristic acid | 3.7023 | 1.8884 | 0.04316 | 1.3649 |
| 92 | 2,4-Di-tert-butylphenol | 9.7436 | 3.2845 | 0.044807 | 1.3486 |
| 93 | 1,4-Dimethoxybenzene | 13.333 | 3.737 | 0.048964 | 1.3101 |
| 94 | Tetradec-2-enal | 13.333 | 3.737 | 0.048964 | 1.3101 |
| 95 | Hexacosanoic acid | 4.2581 | 2.0902 | 0.050681 | 1.2952 |
| 96 | (E,E)-2,4-Decadienal | 9.1667 | 3.1964 | 0.053095 | 1.2749 |
| 97 | 4-Ethyl-2-methoxyphenol | 0.10909 | -3.1964 | 0.053095 | 1.2749 |
| 98 | Palmitelaidic acid | 9.1667 | 3.1964 | 0.053095 | 1.2749 |
| 99 | 20-methyldocosanoic acid | 0.094737 | -3.3999 | 0.05336 | 1.2728 |
| 100 | 2-Decenal | 4.3077 | 2.1069 | 0.055995 | 1.2518 |
| 101 | 5,8,11-Eicosatriynoic acid | 9.4444 | 3.2395 | 0.057893 | 1.2374 |
| 102 | Phthalic acid | 0.4959 | -1.0119 | 0.05858 | 1.2322 |
| 103 | (6E,8E)-4,6,8-Megastigmatriene | 17.5 | 4.1293 | 0.058641 | 1.2318 |
| 104 | 9-Octadecenal | 0.35 | -1.5146 | 0.062241 | 1.2059 |
| 105 | 2-(Octadecyloxy)ethanol | 3.4103 | 1.7699 | 0.063377 | 1.1981 |
| 106 | Cholesta-4,6-dien-3-one | 18.333 | 4.1964 | 0.063905 | 1.1945 |
| 107 | 2'-Hydroxy-5'-methylacetophenone | 0.10909 | -3.1964 | 0.067179 | 1.1728 |
| 108 | Chlordecone | 19.444 | 4.2813 | 0.069115 | 1.1604 |
| 109 | Behenic acid | 0.088889 | -3.4919 | 0.069999 | 1.1549 |
| 110 | D-Fructose | 0.066667 | -3.9069 | 0.072381 | 1.1404 |
| 111 | (E,E)-11,13-Octadecadien-9-ynoic acid | 15 | 3.9069 | 0.072381 | 1.1404 |
| 112 | 3,6-Dimethyl-2(3H)-benzofuranone | 11.667 | 3.5443 | 0.072844 | 1.1376 |
| 113 | Solketal | 11.667 | 3.5443 | 0.072844 | 1.1376 |
| 114 | 2,3,5,8-Tetramethyldecane | 20.833 | 4.3808 | 0.073037 | 1.1365 |
| 115 | xi-3-Methyl-3-cyclohexen-1-ol | 0.05 | -4.3219 | 0.073373 | 1.1345 |
| 116 | Bentonite | 12 | 3.585 | 0.08046 | 1.0944 |
| 117 | 3-Methyl-2,4-nonanedione | 13.333 | 3.737 | 0.082754 | 1.0822 |
| 118 | (E)-3-Octene | 0.03 | -5.0589 | 0.085671 | 1.0672 |
| 119 | 3beta-Ergosta-5,23-dien-3-ol | 11.111 | 3.4739 | 0.090262 | 1.0445 |
| 120 | Aceteugenol | 11.333 | 3.5025 | 0.10314 | 0.98658 |
| 121 | 16b-Hydroxyestradiol | 13.333 | 3.737 | 0.10939 | 0.96101 |
| 122 | 3-Methoxytyramine | 21.667 | 4.4374 | 0.11113 | 0.95418 |
| 123 | Benzene | 0.079832 | -3.6469 | 0.11833 | 0.9269 |
| 124 | (-)-(Z)-Tetrahydro-6-(2-pentenyl)-2H-pyran-2-one | 4.029 | 2.0104 | 0.12178 | 0.91444 |
| 125 | 2,6-Ditert-butyl-4-[(3,5-ditert-butyl-4-hydroxyphenyl)sulfanylmethylsulfanyl]phenol | 54.167 | 5.7593 | 0.12508 | 0.90282 |
| 126 | gamma-Ionone | 4.5 | 2.1699 | 0.13816 | 0.85962 |
| 127 | 10Z-Nonadecenoic acid | 0.066667 | -3.9069 | 0.14073 | 0.85161 |
| 128 | 1-Undecanol | 0.070588 | -3.8244 | 0.14862 | 0.82793 |
| 129 | Sarsasapogenin | 0.2093 | -2.2563 | 0.14932 | 0.82588 |
| 130 | 2-Nonadecanone | 16.667 | 4.0589 | 0.16663 | 0.77826 |
| 131 | 3beta-3-Lupanol | 0.06 | -4.0589 | 0.16663 | 0.77826 |
| 132 | alpha-Carotene | 5.3333 | 2.415 | 0.17135 | 0.76612 |
| 133 | 3-Palmitoyl-sn-glycerol | 2.5778 | 1.3661 | 0.17151 | 0.76572 |
| 134 | 1-Phenylethanol | 0.06 | -4.0589 | 0.2079 | 0.68214 |
| 135 | Geranylgeranyl-PP | 0.39583 | -1.337 | 0.21638 | 0.66479 |
| 136 | Carnosic acid | 0.14286 | -2.8074 | 0.22298 | 0.65173 |
| 137 | Methyl nicotinate | 0.14286 | -2.8074 | 0.22298 | 0.65173 |
| 138 | Pinolidoxin | 8.6667 | 3.1155 | 0.25558 | 0.59247 |
| 139 | Ascaridole | 9.5 | 3.2479 | 0.26735 | 0.57291 |
| 140 | Tridecanedioyl-CoA | 10.333 | 3.3692 | 0.27707 | 0.55741 |
| 141 | 9,12-Octadecadiynoic acid | 65 | 6.0224 | 0.29121 | 0.5358 |
| 142 | 3-(4-Methyl-3-pentenyl)thiophene | 0.017647 | -5.8244 | 0.29964 | 0.5234 |
| 143 | 1-Hexadecanol | 0.071795 | -3.8 | 0.30845 | 0.51082 |
| 144 | 1-hydroxylycopene | 12.7 | 3.6668 | 0.32606 | 0.4867 |

**Table S6. Pathway enrichment analysis of differential metabolites**

| **S.No** | **Pathway name** | **Total** | **Expected** | **Hits** | **Raw p** | **Holm p** | **FDR** |
| --- | --- | --- | --- | --- | --- | --- | --- |
| 1 | Fatty Acid Biosynthesis | 35 | 0.444 | 4 | 0.00066 | 0.0646 | 0.0646 |
| 2 | Beta Oxidation of Very Long Chain Fatty Acids | 17 | 0.216 | 2 | 0.0182 | 1 | 0.891 |
| 3 | Plasmalogen Synthesis | 26 | 0.33 | 2 | 0.0407 | 1 | 1 |
| 4 | Steroid Biosynthesis | 48 | 0.609 | 2 | 0.121 | 1 | 1 |
| 5 | Bile Acid Biosynthesis | 65 | 0.825 | 2 | 0.197 | 1 | 1 |
| 6 | Alpha Linolenic Acid and Linoleic Acid Metabolism | 19 | 0.241 | 1 | 0.217 | 1 | 1 |
| 7 | Mitochondrial Electron Transport Chain | 19 | 0.241 | 1 | 0.217 | 1 | 1 |
| 8 | Tyrosine Metabolism | 72 | 0.914 | 2 | 0.231 | 1 | 1 |
| 9 | Androstenedione Metabolism | 24 | 0.305 | 1 | 0.267 | 1 | 1 |
| 10 | Glycerolipid Metabolism | 25 | 0.317 | 1 | 0.276 | 1 | 1 |
| 11 | Mitochondrial Beta-Oxidation of Medium Chain Saturated Fatty Acids | 27 | 0.343 | 1 | 0.295 | 1 | 1 |
| 12 | Phenylalanine and Tyrosine Metabolism | 28 | 0.355 | 1 | 0.304 | 1 | 1 |
| 13 | Mitochondrial Beta-Oxidation of Long Chain Saturated Fatty Acids | 28 | 0.355 | 1 | 0.304 | 1 | 1 |
| 14 | Urea Cycle | 29 | 0.368 | 1 | 0.313 | 1 | 1 |
| 15 | Starch and Sucrose Metabolism | 31 | 0.394 | 1 | 0.331 | 1 | 1 |
| 16 | Citric Acid Cycle | 32 | 0.406 | 1 | 0.34 | 1 | 1 |
| 17 | Fructose and Mannose Degradation | 32 | 0.406 | 1 | 0.34 | 1 | 1 |
| 18 | Amino Sugar Metabolism | 33 | 0.419 | 1 | 0.348 | 1 | 1 |
| 19 | Fatty Acid Elongation In Mitochondria | 35 | 0.444 | 1 | 0.365 | 1 | 1 |
| 20 | Aspartate Metabolism | 35 | 0.444 | 1 | 0.365 | 1 | 1 |
| 21 | Nicotinate and Nicotinamide Metabolism | 37 | 0.47 | 1 | 0.382 | 1 | 1 |
| 22 | Galactose Metabolism | 38 | 0.482 | 1 | 0.39 | 1 | 1 |
| 23 | Fatty acid Metabolism | 43 | 0.546 | 1 | 0.429 | 1 | 1 |
| 24 | Steroidogenesis | 43 | 0.546 | 1 | 0.429 | 1 | 1 |
| 25 | Arginine and Proline Metabolism | 53 | 0.673 | 1 | 0.501 | 1 | 1 |
| 26 | Warburg Effect | 58 | 0.736 | 1 | 0.534 | 1 | 1 |
| 27 | Purine Metabolism | 74 | 0.939 | 1 | 0.625 | 1 | 1 |
